# Post‐migration psychosocial experiences and challenges amongst LGBTQ+ forced migrants: A meta‐synthesis of qualitative reports

**DOI:** 10.1111/jan.15480

**Published:** 2022-11-01

**Authors:** Maria Gottvall, Calle Brunell, Anna Eldebo, Frida Johansson Metso, Maria Jirwe, Tommy Carlsson

**Affiliations:** ^1^ Department of Health Sciences The Swedish Red Cross University Huddinge Sweden; ^2^ Department of Women's and Children's Health Uppsala University Uppsala Sweden; ^3^ Division of Nursing, Department of Neurobiology, Care Sciences and Society Karolinska Institutet Solna Sweden

**Keywords:** forced migrants, lesbian, gay, bisexual, transgender, and queer (LGBTQ+), meta‐synthesis, psychosocial health, psychosocial support systems, public health nursing, refugees, review, sexual and gender minorities, sexual health

## Abstract

**Aims:**

Synthesize qualitative research to illuminate the post‐migration psychosocial experiences amongst LGBTQ+ forced migrants.

**Design:**

Meta‐synthesis of qualitative reports.

**Data sources:**

Systematic searches in seven databases and manual screenings were performed in July 2021 (21,049 entries screened in total). The final sample included 29 English‐language reports containing empirical qualitative findings about post‐migration experiences and published 10 years prior to the searches, based on migrants as the primary source.

**Review Methods:**

Methodological quality was appraised using the CASP and JBI checklists. Through a collaborative process involving nurse‐midwife researchers and experienced clinical professionals, reports were analysed with a two‐stage qualitative meta‐synthesis including an inductive qualitative content analysis.

**Results:**

The methodological quality was high and the reports included 636 participants in total. Two themes were identified through the meta‐synthesis. The first theme illustrates the psychological distress and numerous challenges and stressors forced migrants face after arrival, including challenges encountered as an LGBTQ+ forced migrant, psychological reactions and manifestations, and practical issues related to resettlement and living conditions. The second theme highlights the resilience and strength they find through various internal processes and external resources, including resilience and strengthening resources, identity formation and establishing and maintaining social relationships.

**Conclusion:**

After arrival in the host country, forced migrants identifying as LGBTQ+ face numerous societal and personal challenges whilst being at risk of experiencing significant psychological distress. These migrants utilize a wide range of resources that may strengthen their resilience. Peer support stands out as a highly appreciated and promising resource that needs further attention in experimental research.

**Impact:**

Forced migrants identifying as LGBTQ+ need access to adequate and sufficient support. The findings emphasize several strength‐building resources that may inform nurses, midwives, researchers and other professionals when providing psychosocial support for these persons.

**Patient or Public Contribution:**

No patient or public contribution.

## 1 INTRODUCTION

Worldwide, the numbers of forced migrants seeking asylum based on persecution, violence and human rights violations are high and increasing; with over 80 million forcibly displaced persons in 2020 (United Nations High Commissioner for Refugees, 2021). Not only does forced migration involves a risk of being exposed to stress before and during the migration, it is also associated with ongoing potential challenges in the host country when seeking asylum and during resettlement (Li et al., 2016). Persecution and personal danger based on diverse sexual orientations, gender identities, and/or gender expressions—herein defined as lesbian, gay, bisexual, transgender, queer, or other non‐heterosexual orientations, non‐cisgender identities, gender expressions and/or reproductive development considered beyond cultural, societal or physiological norms (LGBTQ+) ‐ is acknowledged as a valid reason for being granted asylum (Couldrey & Herson, 2013).

### 1.1 Background

Many countries criminalize diverse sexual orientations, gender identities and/or gender expressions whilst lacking policies that counteract violations of basic human rights (Ramon Mendos et al., 2020). When experiencing inhumane persecution and violence, persons identifying as LGBTQ+ are often forced to flee and seek asylum in other countries. Forced migrants, herein defined as persons who are forced to migrate to another country as a consequence of experiencing their situation in the country of origin as unsafe and threatening, can apply for asylum and resettle in a variety of countries. Although challenging to determine the exact amount of migrants identifying as LGBTQ+, reports suggest a significant number of asylum claims (European Union Agency for Fundamental Rights, 2017).

Being stigmatized, excluded, discriminated, victimized or harassed because of social positioning and/or identity, including when having an LGBTQ+ identity, can result in minority stress. This stress is considered a unique experience for stigmatized or marginalized populations, who encounter continued exposure to socially‐based stressors related to underlying social or cultural structures (Meyer, 2003). Minority stress has been shown to be associated with health‐related consequences (Pitoňák, 2017). Forced migrants are at a high risk of experiencing prolonged and repeated psychological distress when feeling, seeking asylum, and resettling in another country (Nickerson et al., 2011). Intersectionality concerns the interconnectedness of systems of oppression that leads to social inequalities, power and privilege (Collins & Bilge, 2020; Crenshaw, 1989). The concept highlights the importance of understanding multiple and simultaneous disadvantages, which can help inform and improve global health (Kapilashrami & Hankivsky, 2018). Those identifying as LGBTQ+ and are forced migrants have intersecting social or minority identities that shape their situation and can present unique converging stressors. According to previous research, being forced to migrate to another country because of your sexual orientation, gender identity, and/or gender expression is a significant personal undertaking involving substantial risks and health‐related consequences during the transit (Yarwood et al., 2022). These persons are likely confronted with various health‐related challenges in society when seeking asylum and resettling. One scoping review specifically investigating violence and abuse against sexual and gender minority migrants found that they experience new violence and abuse whilst living with past trauma (Alessi, Cheung, et al., 2021). Whilst it is certainly important to address particular challenges in targeted reviews, such as violence and abuse, there is also a need to provide holistic knowledge based on current qualitative evidence. There is a lack of synthesizing review efforts in research aiming to increase the overall understanding of the lived post‐migration experiences in this population. Herein, we broadly define post‐migration as the period beginning when a forced migrant arrives in a host country and has the intention of resettling there. We intended to provide a holistic understanding of the experiences following the arrival, and thus included experiences related to staying in asylum accommodations or other refugee facilities in the host country.

## 2 THE REVIEW

### 2.1 Aims

Given the limited attempts to bring together a synthesis of qualitative research about the experiences of the target population, the overarching aim was to illuminate the post‐migration psychosocial experiences amongst LGBTQ+ forced migrants.

### 2.2 Design

This meta‐synthesis was reported according to the ENTREQ guideline (Supplemental Table S1; Tong et al., 2012). Meta‐synthesis is a systematic approach to provide overarching summaries and integrations of qualitative studies (Sandelowski & Barroso, 2006). This review focuses on qualitative research as naturalistic methodological approaches utilized to explore human experiences and inductively engage in non‐numeric data collection (Patton, 2002).

### 2.3 Search methods

Systematic pre‐planned searches were performed in seven databases in July 2021: CINAHL, Cochrane Library, Open Grey, PsycINFO, PubMed, SCOPUS, and Web of Science. Relevant search terms were identified by exploring keywords in vocabulary thesauruses in the databases and titles/abstracts identified in pilot searches. MeSH‐terms, CINAHL headings, PsycINFO descriptors, Boolean operators, and truncations were used to broaden searches. The final search strings included terms related to migration and LGBTQ+ (Supplemental Table S2). Manual screenings were also performed by inspecting: (1) reference lists in included reports; (2) lists of citing documents in the databases; (3) lists of similar articles in the databases (screening first 1000 entries).

### 2.4 Eligibility criteria

To be included, reports needed to be: (1) a report with qualitative findings distinguishable from other presentations; (2) written in English; (3) published in 10 years; (4) based on LGBTQ+ forced migrants as the primary source of data; and (5) primary empirical research published in a scientific journal. Reports were excluded if: (1) only reporting findings about pre‐ or peri‐migration experiences; (2) only based on secondary sources, or difficult to discern between sources; (3) including non‐forced migrants, or difficult to discern between forced and non‐forced migrants; (4) lacking full‐text documents; or (5) published more than 10 years before searches (Table 1).

**TABLE 1:** Inclusion and exclusion criteria

Domain	Inclusion criteria	Exclusion criteria
Population	Forced migrants self‐identifying as lesbian, gay, bisexual, transgender, queer, or other non‐heterosexual orientations, non‐cisgender identities, gender expressions, and/or reproductive development considered beyond cultural, societal, or physiological norms	Non‐forced migrants; secondary sources of qualitative data
Phenomenon	Post‐migration psychosocial experiences	Pre‐ or peri‐migration experiences
Language	English	Non‐English
Study design	Qualitative research; Mixed‐ or multi‐methods research with clearly distinguishable qualitative analysis and presentation	Quantitative research; Mixed‐ or multi‐methods research with non‐distinguishable qualitative analysis and presentation
Publication time	Published 10 years before searches	Published more than 10 years before searches
Publication type	Primary empirical research published as an article in a scientific journal	Conference proceedings or abstracts; Book chapters; Literature reviews; Letters or editorials; Theses; No full‐text document

### 2.5 Study selection

The last author (TC) screened titles and abstracts, and those fulfilling the inclusion criteria were read as full‐text versions. In ambiguous cases, the first author (MG) read the full‐text report and discussed potential inclusion with the last author until consensus was achieved. Reports were included regardless of methodological quality.

### 2.6 Search outcomes

**FIGURE 1:**
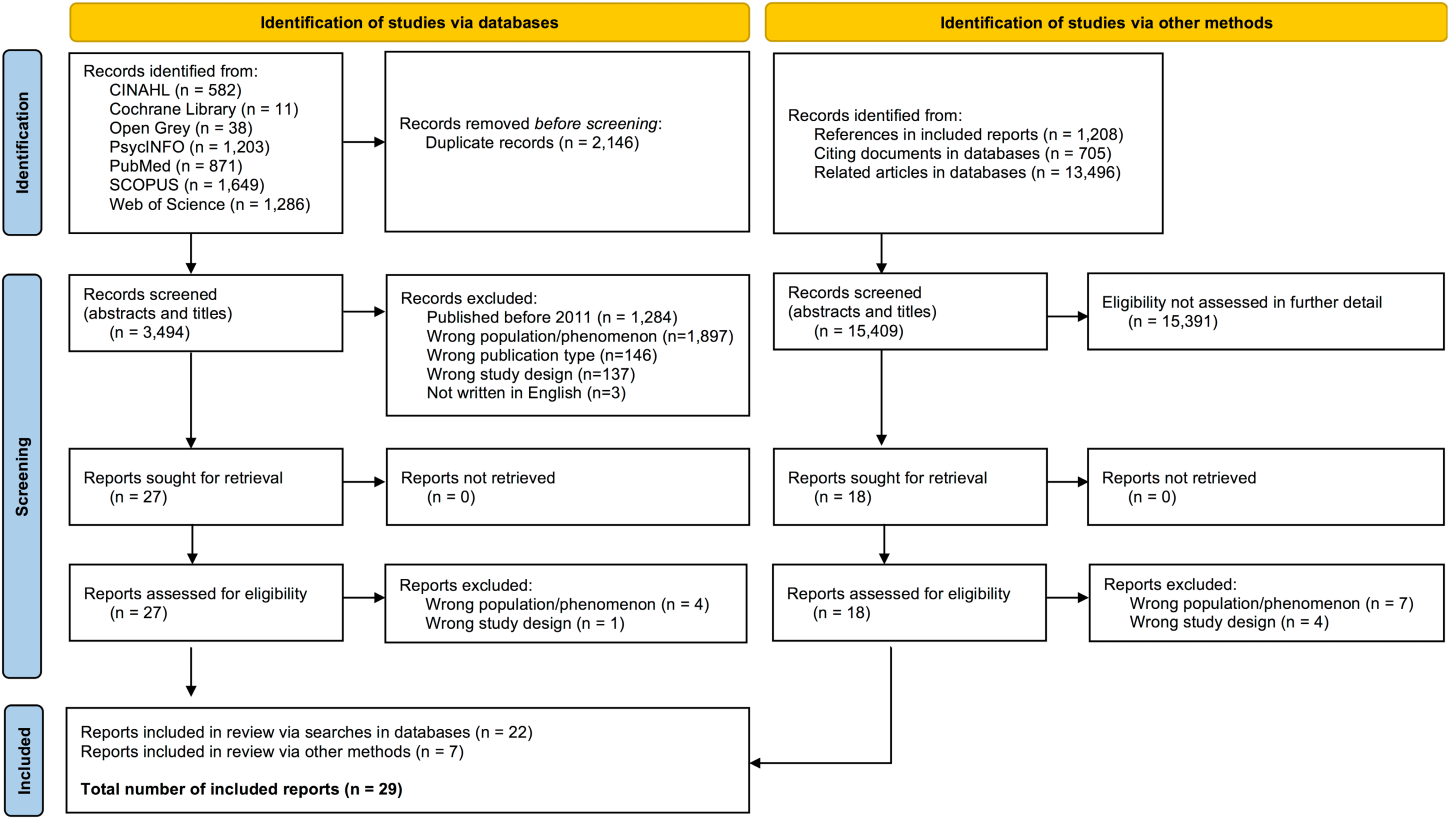
The process of identification and screening of reports.

The systematic searches yielded 5640 hits, of which 2146 were duplicate hits, resulting in 3494 unique entries. In total, 1284 hits led to reports published before 2011, leaving 2210 unique reports. Amongst these, 2183 were excluded because they investigated the wrong population or phenomenon (*n* = 1897), were the wrong publication type (*n* = 146), had the wrong study design (*n* = 137), and were not written in English (*n* = 3). Many reports were excluded in this phase because they did not analyse empirical qualitative research investigating LGBTQ+ forced migrants, for example, reports were excluded based on investigating non‐migrant LGBTQ+ populations or because the document was published as a commentary without any analysis of empirical data. This process resulted in 27 potential reports identified via systematic searches. These were scrutinized by the first (MG) and last (TC) authors, who through joint discussions determined that five would be excluded based on investigating the wrong population or phenomenon (*n* = 4) and having the wrong study design (*n* = 1). This resulted in 22 reports included via systematic searches in databases. A total of 15,409 records were screened through manual searches, including via reference lists (*n* = 1208), citing documents (*n* = 705), and lists of similar articles (*n* = 13,496). Many of these records were discarded because they were not qualitative empirical studies. Following manual searches, the titles and abstracts of 18 reports fulfilled the inclusion criteria. The full‐text documents of the reports were assessed by the first (MG) and last (TC) authors; 11 reports were excluded, resulting in 7 included reports. Thus, 29 reports in total were included in this review (Figure 1).

### 2.7 Data extraction

Methodological details were extracted from all reports by the last author (TC) utilizing a modified version of the JBI Qualitative Data Extraction Tool (Additional File 3) (Lockwood et al., 2020). Ambiguous cases were discussed between the first (MG) and last (TC) authors until a consensus was reached. The texts in sections describing the empirical findings were extracted and analysed.

### 2.8 Quality appraisal

The reporting of methodological quality in the included publications was appraised using the CASP (Critical Appraisal Skills Programme 2018) and the JBI (Lockwood et al., 2020) checklists. The instruments include 10 items, respectively. The first (MG) and last (TC) authors conducted separate appraisals and discussed their findings until a consensus was reached.

### 2.9 Data abstraction and synthesis

Included reports were analysed with the two‐stage qualitative meta‐synthesis process (Sandelowski & Barroso, 2006). Through meta‐synthesis, an abstracted view is shaped in which the findings of several reports are incorporated to form new understandings and insights. In the quantitatively oriented first stage (meta‐summary), the findings in each report were extracted and aggregated to form descriptive renderings. In the more qualitatively oriented second stage (meta‐synthesis), the findings were integrated to form themes.

Inspired by inductive qualitative content analysis (Graneheim & Lundman, 2004), the analytic process involved the following steps: (1) the results of each publication were read carefully to gain an understanding of the overall context and content; (2) meaning units were identified, derived from the extracted results, quotations and paraphrases that spoke of a certain finding; (3) meaning units were condensed and summarized as a code acting as a brief summary; (4) codes that shared a commonality were collated into sub‐categories and categories that illustrated the shared content on a manifest level. To facilitate the researcher‐led manual categorization process, Nvivo version 12.7.0 was utilized.

The first (MG) and last (TC) authors conducted steps 1–4 separately for one report, revealing small insignificant differences. Thus, the remaining reports were divided between the two authors, both registered/specialist nurse‐midwives and researchers with experience in conducting qualitative analyses. All coding procedures were scrutinized through a collaborative process together with the second authors (CB, AE, and FJM), all licensed psychologists with clinical experience in providing support for LGBTQ+ migrants. These provided feedback and aided in the categorization process during several workshops. In line with the meta‐summary approach (Sandelowski & Barroso, 2006), frequency effect sizes (ES) were calculated for each category, i.e., the percentage of reports represented in a certain category.

In the second interpretative phase, categories were abstracted into overarching themes representing a deeper level of interpretation. All authors collaborated in the thematization by carefully inspecting each category and writing comprehensive statements that illustrated all nuances found in the theme. The findings were discussed amongst the authors until consensus was reached and all felt that the synthesis adequately illustrated the findings.

## 3 RESULTS

### 3.1 Quality appraisal and methodological characteristics

**FIGURE 2:**
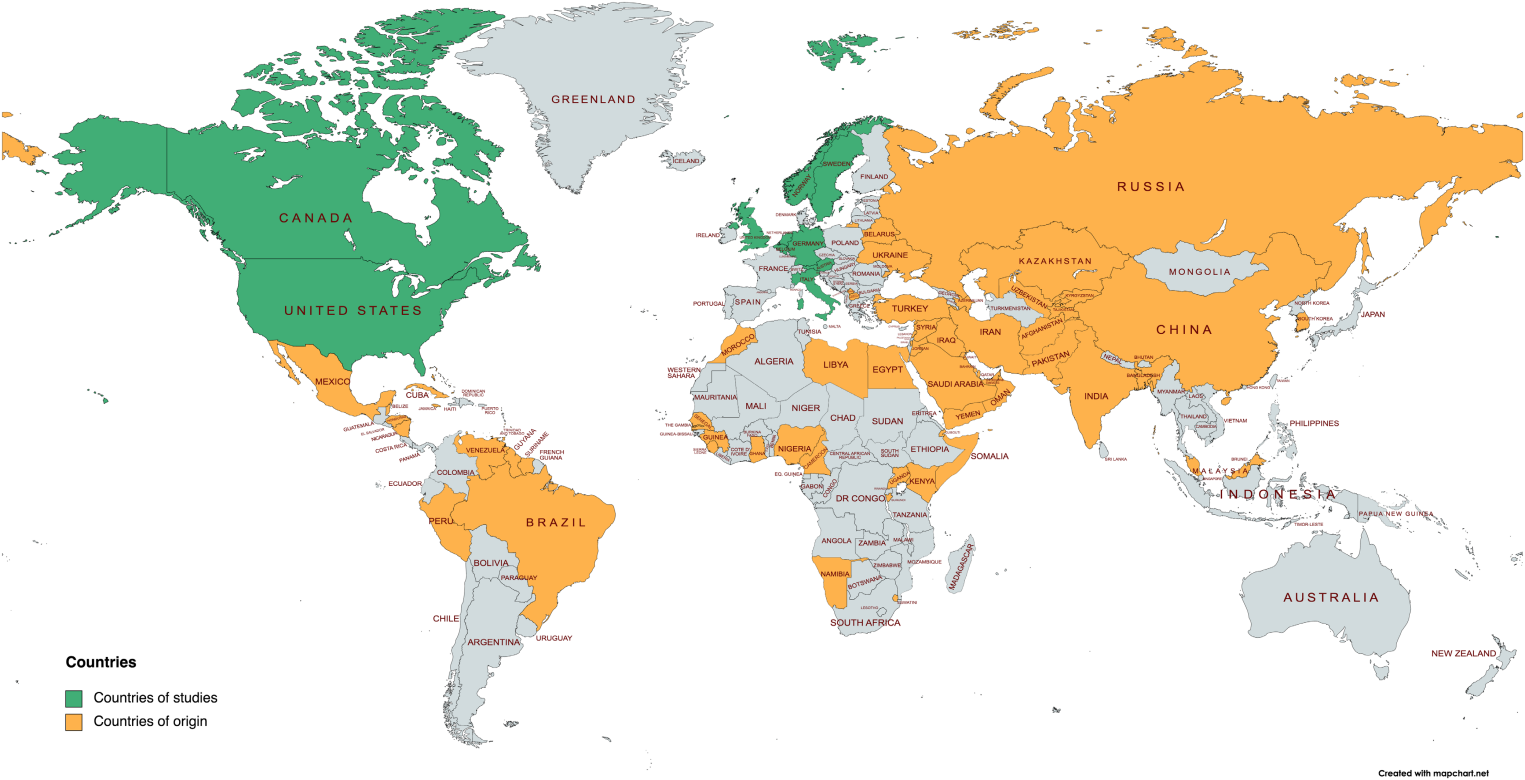
Countries where included reports were conducted and the countries of origin amongst the participants in the reports, when specified (created with mapchart.net).

The methodological quality was generally acceptable, with a majority of the reports adhering to six or more of the criteria in the CASP (*n* = 24, 83%) and JBI (*n* = 24, 83%) checklists (Table 2). All studies were conducted between 2008 and 2020 in North America or Europe (Table 3, Figure 2, and Additional File 4). The most utilized methods were snowball sampling, interviews, and thematic analysis. The reports included 636 participants in total originating from seven regions, most common being the Middle East and Africa (Table 4 and Figure 2). Cisgender was the most common gender identity and the most represented sexual orientation was gay/men who desire men. Varied migration statuses were represented, most common being granted asylum, permanent residence or recognized refugee.

**TABLE 2:** Methodological appraisal of the reports (*n* = 29)

Checklist	Yes, *n* (%)	No, *n* (%)	Unclear, *n* (%)
CASP checklist			
Clear statement of the aims	28 (97%)	—	1 (3%)
Qualitative methodology appropriate	29 (100%)	—	—
Research design appropriate to address the aims	29 (100%)	—	—
Recruitment strategy appropriate to the aims	22 (76%)	—	7 (24%)
Data collected in a way that addressed research issue	24 (83%)	—	5 (17%)
Researcher/participant relationship considered	10 (35%)	1 (3%)	18 (62%)
Ethical issues taken into consideration	20 (69%)	—	9 (31%)
Data analysis sufficiently rigorous	17 (59%)	—	12 (41%)
Clear statement of findings	26 (90%)	1 (3%)	2 (7%)
JBI checklist			
Congruity between philosophical perspective and methodology	28 (97%)	—	1 (3%)
Congruity between methodology and research objectives	26 (90%)	—	3 (10%)
Congruity between methodology and methods to collect data	25 (86%)	—	4 (14%)
Congruity between methodology and representation, analysis	18 (62%)	—	11 (38%)
Congruity between methodology and interpretation	25 (86%)	—	4 (14%)
Statement locating researcher culturally/theoretically	9 (31%)	17 (59%)	3 (10%)
Researcher influence on research, and vice‐versa, is addressed	13 (45%)	14 (48%)	2 (7%)
Participants, and their voices, are adequately represented	26 (90%)	—	3 (10%)
Ethical according to current criteria and ethics approval	20 (69%)	—	9 (31%)
Conclusions flow from analysis, or interpretation, of data	22 (76%)	—	7 (24%)

**TABLE 3:** Methodological characteristics of the reports (*n* = 29)

Characteristic		Reports, *n* (%)
Participant recruitment	Snowball sampling	14 (48%)
	Purposeful sampling	10 (34%)
	Convenience sampling	9 (31%)
	Sampling not specified	5 (17%)
Data collection	Individual interviews	27 (93%)
	Focus group discussions	4 (14%)
	Observations	2 (7%)
Region study was conducted	North America	17 (59%)
	Europe	12 (41%)
Analysis	Thematic analysis	13 (45%)
	Grounded theory/constant comparative analysis	6 (21%)
	Ethnographic analysis/ethnographic fieldwork	3 (10%)
	Interpretative phenomenology analysis	2 (7%)
	Case analysis	1 (3%)
	Narrative thematic analysis	1 (3%)
	Participatory action research	1 (3%)
	Analysis not specified	7 (24%)

**TABLE 4:** Characteristics of the participants (*n* = 636) in the reports (*n* = 29)

Characteristic	Participants, *n* (%)	Reports, *n* (%)
Region of origin		
Middle East	181 (28%)	23 (79%)
Africa	93 (15%)	21 (72%)
Caribbean	71 (11%)	10 (34%)
Asia	46 (7%)	13 (45%)
Russia or Chechnya	23 (4%)	10 (34%)
Latin America	16 (3%)	8 (28%)
Europe	5 (1%)	4 (14%)
Region of origin not presented^a^	201 (32%)	12 (41%)
Gender identity^b^		
Cisgender men^c^	253 (40%)	16 (55%)
Cisgender women^c^	71 (11%)	7 (24%)
Transgender women	40 (6%)	9 (31%)
Gender queer, gender nonconforming or non‐binary	16 (3%)	6 (21%)
Transgender (not specified in further detail)	10 (2%)	6 (21%)
Transgender men	5 (1%)	4 (14%)
Two‐spirit	2 (0.3%)	1 (3%)
Bi‐gender	1 (0.2%)	1 (3%)
Gender not presented^a^	271 (43%)	10 (34%)
Sexual orientation^b^		
Gay and men who desire men	314 (49%)	23 (79%)
Bisexual	53 (8%)	10 (34%)
Lesbian	52 (8%)	13 (45%)
Heterosexual	12 (2%)	4 (14%)
Queer	4 (1%)	2 (7%)
Pansexual	2 (0.3%)	2 (7%)
Described as other (not specified in further detail)	1 (0.2%)	1 (3%)
Sexual orientation not presented^a^	224 (35%)	16 (55%)
Migration status		
Granted, permanent residence, or recognized refugee	143 (22%)	12 (41%)
Seeking asylum, asylum applicant, or asylee	102 (16%)	10 (34%)
Denied asylum, undocumented, or overstay	15 (2%)	7 (24%)
Subsidiary protection or deportation ban	8 (1%)	2 (7%)
Withholding of removal	2 (0.3%)	1 (3%)
Temporary status granted	2 (0.3%)	2 (7%)
Visa	2 (0.3%)	1 (3%)
Migration status not presented^a^	362 (57%)	15 (52%)

^a^ Refers to either the total sample or a subsample.

^b^ Possible for the same participant to have several gender identities or sexual orientations.

^c^ Categorized as cisgender when the report either stated cisgender specifically or stated gender as men or women without mentioning transgender.

### 3.2 Meta‐synthesis of qualitative findings

**FIGURE 3:**
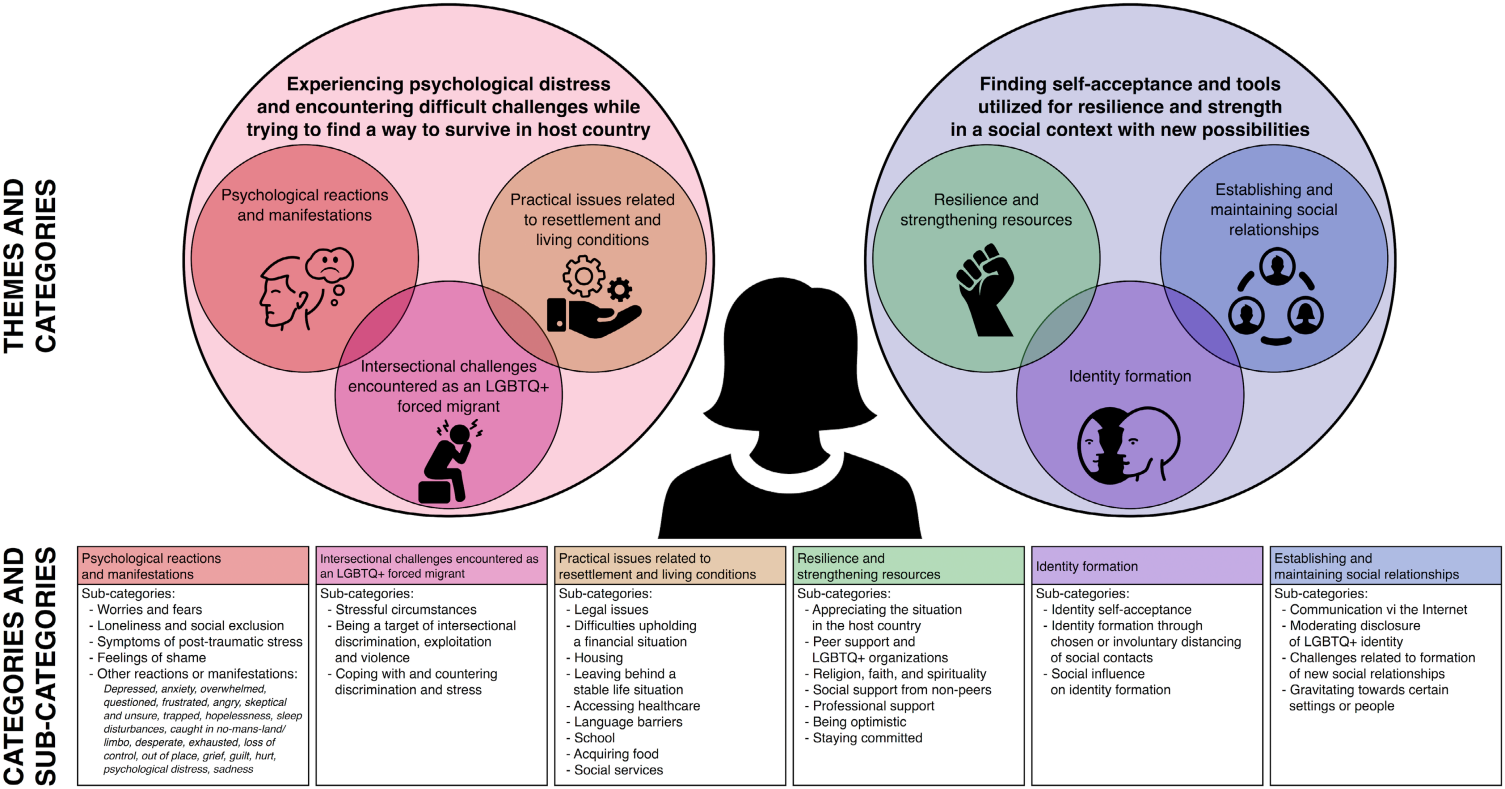
Identified themes, categories, and sub‐categories.

Two larger themes were identified, illustrating the significant psychological distress and stressors migrants face, whilst simultaneously highlighting the resilience and strength they find through numerous internal processes and external resources (Figure 3). For a detailed presentation of references, illustrative quotes, and frequency effect sizes, please see Additional Files 5 and 6.

#### 3.2.1 Theme 1: Experiencing psychological distress and encountering difficult challenges whilst trying to find a way to survive and live in the host country

The theme contains three categories: *intersectional challenges encountered as an LGBTQ+ forced migrant* (ES 97%; Akin, 2017; Alessi, 2016; Alessi et al., 2018, 2020; Alessi, Greenfield, et al., 2021; Cerezo et al., 2014; Dhoest, 2019, 2020; Golembe et al., 2020; Held, 2022; Kahn, 2015a, 2015b; Kahn & Alessi, 2018; Karimi, 2020a, 2020b, 2021; Kostenius et al., 2021; Lee & Brotman, 2011; Llewellyn, 2021; Logie et al., 2016; Mulé, 2021; Murray, 2014a, 2014b; Novitskaya, 2021; Oren & Gorshkov, 2021; Rosati et al., 2021; Wimark, 2019, 2021), *psychological reactions and manifestations* (ES 86%; Akin, 2017; Alessi, 2016; Alessi et al., 2018, 2020; Cerezo et al., 2014; Golembe et al., 2020; Held, 2022; Kahn, 2015a, 2015b; Kahn et al., 2018; Kahn & Alessi, 2018; Karimi, 2020a, 2021; Kostenius et al., 2021; Lee & Brotman, 2011; Llewellyn, 2021; Logie et al., 2016; Mulé, 2021; Murray, 2014a, 2014b; Oren & Gorshkov, 2021; Rosati et al., 2021; Wimark, 2019, 2021), and *practical issues related to resettlement and living conditions* (ES 66%; Akin, 2017; Alessi, 2016; Alessi et al., 2018, 2020; Cerezo et al., 2014; Golembe et al., 2020; Kahn, 2015a; Kahn & Alessi, 2018; Karimi, 2020a; Lee & Brotman, 2011; Llewellyn, 2021; Logie et al., 2016; Mulé, 2021; Murray, 2014a, 2014b; Novitskaya, 2021; Oren & Gorshkov, 2021; Wimark, 2019).

##### Intersectional challenges encountered as an LGBTQ+ refugee (ES 97%)

Subtle and overt forms of discrimination, marginalization and social exclusion related to racism, transphobia and homophobia permeated the lives of migrants. Often, these occurrences were based on their intersecting identities, including ethnicity, sexual orientation and gender identity. Migrants were targets of repeated intense violence and threats, involving serious danger and distress. Persons in the host country exploited and exotified them, including expecting sexual acts for favours. Migrants also experienced social exclusion, alienation, and marginalization in communities and public spaces geared towards homonormative, cisgender, and/or white persons. A range of different perpetrators exposed them to discrimination and violence. Some described worse discrimination than before migrating, with daily experiences and discrimination encountered everywhere in society. Various strategies were utilized to cope and counter discrimination, most prominently concealing their LGBTQ+ identity or distancing from the LGBTQ+ scene. Participants staying in asylum accommodations described a significant need to conceal their LGBTQ+ identity from other compatriots. Despite their efforts, migrants expressed few or no ways to counter discrimination and violence. Some utilized harmful coping strategies, including substance abuse or self‐harm.

Asylum hearings represented a considerable challenge when having to collect evidence, repeatedly retell traumatic experiences, and describe intimate details. This was further compounded when experiencing disbelief amongst caseworkers and having to conform to expected stereotypes. Those experiencing rapid asylum processes felt pressured to talk when not feeling ready to do so, whilst long processes involved precariousness, uncertainty and frustration. Migrants also experienced significant distress when living in asylum accommodations, being forced to share living spaces together with hostile and discriminating refugees. Additional stressful circumstances included living unemployed, being placed in detention, and needing to wait a long time for work permits. Some experienced persistent grief when previous relationships were ruptured or relinquished because of their LGBTQ+ identity.

##### Psychological reactions and manifestations (ES 86%)

A range of psychological reactions and manifestations were described. The most prominent were worries and fears of what their future would entail, including the risk of being deported, the risk of being exposed to discrimination and violence and the possible consequences of living openly as LGBTQ+. Migrants feared not having adequate protection and rights, not finding affirming health professionals, and losing their ethnic identity or their understanding of themselves. Another significant challenge was loneliness and social exclusion—which was particularly prominent when living in asylum accommodations, interacting in queer communities, waiting for the asylum decision and living undocumented. Insufficient language skills, limited social networks, not feeling ready to tell their stories, and being unemployed further contributed to social exclusion. Some became socially isolated because of mental exhaustion.

Symptoms of post‐traumatic stress were described in seven reports, often triggered by asylum hearings when prompted to retell memories and previous exposure to trauma in their country of origin. Feelings of shame also manifested amongst migrants; related to previous exposure to trauma, their sexual orientation and desires, relationships, their bodies, needing mental health services, and need to wait long periods for work permits. A range of other psychological reactions and manifestations were also described, with the highest ES for feeling depressed, experiencing anxiety and feeling overwhelmed.

##### Practical issues related to resettlement and living conditions (ES 66%)

Migrants needed to deal with various practical issues related to resettlement and living conditions. Dealing with legal issues was necessary when applying for asylum, which involved difficulties securing the needed representation whilst being tasked to provide a convincing case. Some encountered disbelief and poor translation/interpretation services, hindering a successful hearing. To strengthen their claims, migrants felt a need to deepen their connections with the LGBTQ+ community, and to go public about their stories. They also felt a need to change their behaviours according to expected stereotypes of LGBTQ+. As a last resort, some contemplated on producing photos of sexual acts as an attempt to enhance their credibility in the asylum‐claiming process and prove their sexual orientation.

Several reports described difficulties upholding a financial situation. Securing employment was a demanding process, largely because of intersectional bias and discrimination when interacting with employers. The financially unstable situation led migrants into involuntary work, including engaging in involuntary sex work or unwanted relationships. The need to wait long periods before obtaining work permits further contributed to their status as unemployed. Transgender migrants had to deal with conflicting documents when their gender identity and pronouns did not match their names in official documents.

Migrants needed to ensure that their basic daily needs for safe housing were met, which was related to complex challenges and the risk of becoming homeless. This included experiencing threatening, controlling, discriminating, and questioning behaviours amongst landlords, needing to conceal their LGBTQ+ identity and difficulties attaining affordable housing. Not having adequate access to basic healthcare services was also experienced, including difficulties navigating the health care system and not understanding which services that were covered. This further contributed to poor health and financial instability. Practical challenges were also experienced related to language barriers, not being able to apply for education, difficulties acquiring sufficient food, and not having access to adequate social services.

#### 3.2.2 Theme 2: Finding self‐acceptance and tools utilized for resilience and strength in a social context with new possibilities

The theme contains three categories: *resilience and strengthening resources* (ES 90%) (Alessi, 2016; Alessi et al., 2018, 2020; Alessi, Greenfield, et al., 2021; Cerezo et al., 2014; Dhoest, 2019, 2020; Golembe et al., 2020; Held, 2022; Kahn, 2015a, 2015b; Kahn et al., 2018; Karimi, 2020a, 2020b, 2021; Kostenius et al., 2021; Lee & Brotman, 2011; Logie et al., 2016; Mulé, 2021; Murray, 2014a, 2014b; Novitskaya, 2021; Oren & Gorshkov, 2021; Rosati et al., 2021; Wimark, 2019, 2021), *identity formation* (ES 62%; Akin, 2017; Alessi, Greenfield, et al., 2021; Cerezo et al., 2014; Dhoest, 2020; Golembe et al., 2020; Held, 2022; Kahn, 2015a, 2015b; Karimi, 2020a, 2020b, 2021; Lee & Brotman, 2011; Llewellyn, 2021; Logie et al., 2016; Murray, 2014a; Oren & Gorshkov, 2021; Rosati et al., 2021; Wimark, 2019), and *establishing and maintaining social relationships* (ES 45%; Dhoest, 2019, 2020; Held, 2022; Kahn, 2015a; Karimi, 2020a, 2020b, 2021; Mulé, 2021; Murray, 2014a, 2014b; Oren & Gorshkov, 2021; Wimark, 2019, 2021).

##### Resilience and strengthening resources (ES 90%)

Migrants expressed great appreciation for the opportunity to live as their authentic selves, which resulted in relief, excitement, optimism, freedom, and self‐acceptance. Through the increased legal protection and living in a social context allowing LGBTQ+ identities, they could lead a life without the same strong fear of harassment and oppression as experienced in their country of origin. When contrasting pre‐migration and post‐migration hardships, migrants described an improved overall situation.

A significant contributor to resilience and strength was peer support, as migrants developed meaningful and strong relationships with like‐minded and understanding companions in a safe and accepting setting. Through peer support, they established collective trust and a social setting where they belonged ‐ breaking social isolation, exclusion, and structural marginalization. When seeing peers thrive and meeting role models, migrants felt less fearful and more hopeful. Peer support led to migrants feeling strengthened and better equipped to push back structural barriers. It improved mental health, including enhanced confidence, self‐worth and identity acceptance, whilst reducing feelings of shame, depression, and loneliness. Interacting with peers involved the exchange of emotional support (contributing to feeling normalized, less stressed, valued, and relief), informational support (sharing valuable information and knowledge), and instrumental peer support with practical aid (such as financial help and housing). Providing peer support and taking part in activism was rewarding and a driving force for enhanced resilience, involving an opportunity to transcend their own situation and being a distraction from their own hardships. Engaging in volunteer work and activism was seen as a meaningful endeavour, feeling productive whilst contributing to social change and awareness. However, some felt unaware of peer support organizations or declined a need for peer activities. Some described negative effects on mental health when spending time with peers and LGBTQ+ organizations (which is elaborated on in the next sections).

Another source of resilience was social support from non‐peers in the host community. Receiving this support resulted in mixed feelings, involving both positive experiences and appreciation but also feeling dependent and exposed to potential exploitation and abuse. Migrants formed new chosen families and found significant others, associated with personal benefits and improved quality of life. Some were able to find ways to coexist and receive support from their co‐ethnic communities. Professional support from mental health services offered important tools for psychological adaptation and relief. However, some emphasized the importance of being able to connect with and trust their counsellor. Coming in contact with adequate services was challenging when unaware of available services or if deciding not to seek out professional support because of shame or fear of stigma. Migrants also received support from lawyers and social service workers. Transgender migrants expressed a fundamental role in gaining access to gender affirmation services.

Religion, faith and spirituality could bolster resilience and enhance self‐acceptance. Migrants turned to faith to persevere and cope with discrimination and bias. In some cases, the need to attend religious locations such as churches preceded their need to be open about their LGBTQ+ identity. At times, religious migrants were welcomed in these types of locations whilst living an open LGBTQ+ lifestyle. Migrants also emphasized the importance of remaining hopeful during long asylum processes, thinking optimistically about their future and not dwelling on their past, even when their situation meant a loss of dreams and aspirations or despite feeling isolated and dehumanized. They were committed to taking jobs, invested much time in securing employment, and kept a resourceful attitude.

##### Identity formation (ES 62%)

This category concerns the described internal processes in the participants throughout their journeys of potential self‐acceptance or realization of their identity as well as internal coping strategies utilized by some migrants. Some migrants expressed self‐acceptance following their arrival, acknowledging all parts of their identity without labels and not feeling scared whilst allowing their identity to change over time. Feeling free to express themselves, some decided to live ‘loud and proud’, embracing a public lifestyle not previously possible. Migrants also reconciled their views of religion and faith; some decided to shun the religious doctrines whilst others redefined it in their own terms. In contrast, other migrants did not describe a journey towards self‐acceptance. A strategy to avoid negative social consequences and achieve a higher level of personal freedom was to restrict their communication with ‐ or decide to altogether shun away from ‐ heteronormative compatriots, family members, and relatives. Some also distanced themselves from news related to their country of origin or were reluctant to join LGBTQ+ activities, organizations and/or persons. Distancing from heterosexual persons, in general, was also represented amongst migrants. Migrants found themselves linked with both their old and new social structures, as they had ties, responsibilities, and feelings towards family and friends in their country of origin whilst resettling in the host country. Social and cultural forces further were described as having an influence on the conceptualization of sexual orientation and gender identity.

##### Establishing and maintaining social relationships (ES 45%)

After their arrival, new social relationships that strengthened resilience were formed whilst social relationships already formed were maintained. However, complex social challenges and close‐mindedness were encountered when trying to manage communication and forming meaningful relationships. Migrants balanced interacting with both phobic and affirmative, as well as coethnic and racist, members in various communities. Some LGBTQ+ spaces and peer support groups led by white persons were experienced as being permeated by power differentials and oppressive structures. Another issue raised by migrants was that LGBTQ+ organizations sometimes focused too heavily on newcomers and emergency help. Socioeconomic barriers, expectations on how to act as an LGBTQ+ person, and a lack of interest amongst non‐migrants further hindered the formation of social relationships. Following their arrival, migrants gained access to online communication possibilities and information previously less available in their countries of origin. The Internet was used to stay in touch with persons and communities in their country of origin as well as to establish new contacts in the host country.

Migrants expressed varied preferences about the setting they wanted to live in and whom they wanted to form relationships with. Some gravitated towards larger urban areas where they felt safer and had easier access to LGBTQ+ communities. Others preferred rural areas, as densely populated settings induced discomfort and stress. A preference towards older persons and LGBTQ+ migrants was also expressed. To keep their relationships intact, some moderated what they disclosed on social media and how they justified their emigration, being careful to not make their LGBTQ+ identity visible.

## 4 DISCUSSION

This review synthesized qualitative research about post‐migration psychosocial experiences amongst LGBTQ+ forced migrants. Two overarching themes were identified, portraying the challenges and struggles encountered in the host country as well as the internal and external resources building resilience and strength.

An important finding was the significant and inhumane discrimination, violence, and exploitation based on intersectional identities experienced in the host country. Research shows that LGBTQ+ people and migrants in general experience discrimination, influenced by their intersecting identities (Vaccaro & Koob, 2019). Not only did the participants in the reports encounter systematic exclusion and victimization in society, but they also experienced significant resistance, racism and LGBTQ+ phobia in both queer and co‐ethnic communities. Similar findings have been reported in other reviews highlighting microaggressions and violence enacted by co‐ethnic communities and mainstream white LGBTQ+ spaces, leading to daily challenges, social exclusion, serious health‐related consequences, and risk‐taking behaviours (Alessi, Cheung, et al., 2021; Haghiri‐Vijeh & Clark, 2022; Sadika et al., 2020). In itself, forced migration and having to seek asylum is a life‐changing events associated with considerable psychological sequelae (Li et al., 2016; Yarwood et al., 2022). Our findings confirm that the target population not only experience a range of general challenges in the post‐migration period—regardless of their sexual orientation, gender identity and/or gender expression—but also faces specific burdens related to their multiple intersecting identities, orientations, and social positions. These findings build on and confirm the theories on minority stress (Pitoňák, 2017) and intersectionality (Crenshaw, 1989), acknowledging them as a population at risk of health‐related consequences that need to be adequately acknowledged and approached. Health professionals, leaders, researchers and health educators have an important obligation to work towards inclusion in health (International Council of Nurses, 2021).

Peer support emerged as a valued resource leading to increased strength and resilience. As a concept, peer support through social networks involves the provision of emotional, appraisal, instrumental, and informational support facilitated by persons sharing similar experiences or identities (Dennis, 2003), which was confirmed by our findings. Peer support occurred frequently amongst these migrants who seek out peers they can relate to. Whilst previous reviews have identified peer support as having potential positive and protective effects for persons identifying as LGBTQ+, they also call attention to the need for more research to reach firmer conclusions about its significance and function (Kia et al., 2021). Specifically, in relation to an intersectional approach taking ethnicity into account (Dowers et al., 2020). There is a lack of research interventions evaluating the effects of peer support amongst LGBTQ+ forced migrants. Our findings indicate that peer support is a potentially promising intervention that may result in a range of positive health‐related outcomes. Migrants described psychological distress when experiencing loneliness and social exclusion; which at times was exacerbated when living in remote locations such as rural asylum accommodations. It is possible that a peer support intervention via the Internet would be a suitable approach to accommodate this problem. We highly encourage researchers to conduct trials investigating online peer support.

### 4.1 Implications for practice

The findings illustrate the importance of applying a person‐centred approach during clinical encounters, without being constrained by norms or ideals. Although none of the included studies specifically focused on nursing or midwifery, our results are nevertheless applicable and relevant in clinical praxis in which professionals support these migrants. Nurses and midwives should take into consideration the numerous challenges these migrants encounter in the host country. We encourage more research about LGBTQ+ forced migrants in the field of nursing and midwifery. The significant psychological distress and the potential cumulative effects experienced amongst these migrants calls attention to the importance of adequate routines for screening procedures. Professionals need to ask about experiences related to violence and discrimination in the host country, and when needed, be prepared to inform patients about their rights for protection as well as refer them to other services. Professionals should consider presenting peer support as a strengthening activity, and when appropriate, inform migrants about available organizations that provide social support services, including peer support. Public policy is essential to achieve improved health and well‐being in marginalized populations in society. The recent global pandemic has highlighted important health inequalities encountered by LGBTQ+ forced migrants, illustrating the need for an intersectional approach when analysing vulnerabilities during human crises (Reid & Ritholtz, 2020).

### 4.2 Limitations

There are methodological aspects that need to be considered. First, two of the authors were involved in the identification of reports (MG and TC). Ambiguous cases were settled through discussions until a consensus was reached. We acknowledge that it is possible that we could have dismissed some relevant reports during the screening procedure or because of limitations related to the chosen search terms. Further, this review aimed to synthesize empirical reports in peer‐reviewed scientific journals. We included scientific reports in English published in 10 years, which could limit the number of reports finally included. It is possible that including other literature such as theses written in other languages would have generated different findings. On the other hand, we utilized several databases and screened a larger amount of entries through systematic processes. Many entries were duplicates, indicating saturation in the screening procedure. Second, the methodological appraisal was conducted by two of the authors (MG and TC) and—whilst we do argue that it is a strength that we settled conflicts through discussion—it is nevertheless possible that our biases affected these appraisals. Third, the included reports represent a limited variation in regard to countries where the studies were conducted. We encourage researchers to conduct qualitative research in other settings, to expand the field and increase the potential of transferability. Further, a proportion of the reports did not include specific information concerning how the legal status and/or LGBTQ+ identity of participants related to their experiences. This made it difficult to synthesize specific findings about subgroups and we encourage researchers to include more details in future studies. Forth, a large majority of the participants were cisgender gay men. Although a few reports specifically investigated transgender persons, more qualitative research is needed that explores lived experiences amongst a wider variety of persons in the LGBTQ+ community, including transgender migrants. We involved nurse‐midwives, senior researchers, and licensed psychologists with clinical experience in supporting these migrants through a collaborative approach during the thematic analysis. Whilst findings based on qualitative analyses are always, to some degree, influenced by analyst biases and preconceptions, we argue that the utilization of several analysts, bringing a variety of perspectives, enhances the neutrality of our findings. Whilst we were not guided by thematic saturation, we did nonetheless note a tendency of saturation during the analysis.

## 5 CONCLUSION

Following their arrival in the host country, forced migrants identifying as LGBTQ+ face numerous societal and personal challenges related to their intersecting identities whilst being at risk of experiencing significant psychological distress. Forced migrants identifying as LGBTQ+ need access to adequate and sufficient support. The findings emphasize several resources that may strengthen and build resilience amongst migrants, which can inform nurses, midwives, researchers, and other professionals providing psychosocial support. Peer support stands out as a highly appreciated and promising intervention that needs further attention in research.

## AUTHOR CONTRIBUTIONS

Maria Gottvall: conceptualization, screening of reports, data extraction, methodological appraisal, thematic synthesis, and writing—original draft. Calle Brunell: thematic synthesis and writing—review and editing. Anna Eldebo: thematic synthesis and writing—review and editing. Frida Johansson Metso: thematic synthesis and writing—review and editing. Maria Jirwe: conceptualization and writing—review and editing. Tommy Carlsson: conceptualization, screening of reports, data extraction, methodological appraisal, thematic synthesis, visualization, writing—original draft, supervision, and project administration. All authors approved the final version of the manuscript.

## FUNDING INFORMATION

This research was funded in whole or in part by Forte [GD‐2021/0028].

## CONFLICT OF INTEREST

The authors declare no conflicts of interest.

### PEER REVIEW

The peer review history for this article is available at https://publons.com/publon/10.1111/jan.15480.

## Supporting information


File 1

File 2

File 3

File 4

File 5

File 6

## Data Availability

The data that supports the findings of this study are available in the supplementary material of this article.
