# Supplementary material for: Post‐migration psychosocial experiences and challenges amongst LGBTQ+ forced migrants: A meta‐synthesis of qualitative reports
Source: J Adv Nurs. 2022 Nov 1;79(1):358–71. doi: 10.1111/jan.15480 (PMC10092230; doi:10.1111/jan.15480)
Supplement: Supplementary file 1 — File 1 [file JAN-79-358-s002.pdf]

Additional File 1. The ENTREQ checklist.

Item	Guide and description	Reported on page #
Aim	State the research question the synthesis addresses	3
Synthesis methodology	Identify the synthesis methodology or theoretical framework which underpins the synthesis, and describe the rationale for choice of methodology (e.g., meta-ethnography, thematic synthesis, critical interpretive synthesis, grounded theory synthesis, realist synthesis, meta-aggregation, meta-study, framework synthesis).	4
Approach to searching	Indicate whether the search was pre-planned (comprehensive search strategies to seek all available studies) or iterative (to seek all available concepts until they theoretical saturation is achieved).	4
Inclusion criteria	Specify the inclusion/exclusion criteria (e.g., in terms of population, language, year limits, type of publication, study type).	4
Data sources	Describe the information sources used (e.g. electronic databases (MEDLINE, EMBASE, CINAHL, psycINFO, Econlit), grey literature databases (digital thesis, policy reports), relevant organisational websites, experts, information specialists, generic web searches (Google Scholar) hand searching, reference lists) and when the searches conducted; provide the rationale for using the data sources.	4
Electronic Search strategy	Describe the literature search (e.g. provide electronic search strategies with population terms, clinical or health topic terms, experiential or social phenomena related terms, filters for qualitative research, and search limits).	4, Additional File 2
Study screening methods	Describe the process of study screening and sifting (e.g. title, abstract and full text review, number of independent reviewers who screened studies).	4-5
Study characteristics	Present the characteristics of the	6, Table 3, Additional File 3

	included studies (e.g., year of publication, country, population, number of participants, data collection, methodology, analysis, research questions).	
Study selection results	Identify the number of studies screened and provide reasons for study exclusion (e.g., for comprehensive searching, provide numbers of studies screened and reasons for exclusion indicated in a figure/flowchart; for iterative searching describe reasons for study exclusion and inclusion based on modifications t the research question and/or contribution to theory development).	4-5, Figure 1
Rationale for appraisal	Describe the rationale and approach used to appraise the included studies or selected findings (e.g., assessment of conduct (validity and robustness), assessment of reporting (transparency), assessment of content and utility of the findings).	4-5
Appraisal items	State the tools, frameworks and criteria used to appraise the studies or selected findings (e.g., Existing tools: CASP, QARI, COREQ, Mays and Pope [25]; reviewer developed tools; describe the domains assessed: research team, study design, data analysis and interpretations, reporting).	5
Appraisal process	Indicate whether the appraisal was conducted independently by more than one reviewer and if consensus was required.	5
Appraisal results	Present results of the quality assessment and indicate which articles, if any, were weighted/excluded based on the assessment and give the rationale.	4, 6
Data extraction	Indicate which sections of the primary studies were analysed and how were the data extracted from the primary studies? (e.g., all text under the headings "results /conclusions" were extracted electronically and entered into a	5

	computer software).	
Software	State the computer software used, if any.	5
Number of reviewers	Identify who was involved in coding and analysis.	5-6
Coding	Describe the process for coding of data (e.g., line by line coding to search for concepts).	5-6
Study comparison	Describe how were comparisons made within and across studies (e.g., subsequent studies were coded into pre-existing concepts, and new concepts were created when deemed necessary).	5-6
Derivation of themes	Explain whether the process of deriving the themes or constructs was inductive or deductive.	5-6
Quotations	Provide quotations from the primary studies to illustrate themes/constructs, and identify whether the quotations were participant quotations of the author's interpretation.	Additional Files 4 and 5
Synthesis output	Present rich, compelling and useful results that go beyond a summary of the primary studies (e.g., new interpretation, models of evidence, conceptual models, analytical framework, development of a new theory or construct).	6-11
