# Supplementary material for: Post‐migration psychosocial experiences and challenges amongst LGBTQ+ forced migrants: A meta‐synthesis of qualitative reports
Source: J Adv Nurs. 2022 Nov 1;79(1):358–71. doi: 10.1111/jan.15480 (PMC10092230; doi:10.1111/jan.15480)
Supplement: Supplementary file 2 — File 2 [file JAN-79-358-s006.pdf]

Additional File 2. Search procedure in utilized databases.

Searches were performed and hits were retrieved in 21-07-2021

Pubmed
(("Sexual and Gender Minorities"[Mesh]) OR (queer*) OR (homosexual*) OR (”sexual minorit*”) OR (”sexual orientation*”) OR (”gender minorit*”) OR (lesbian*) OR (gay) OR (bisexual*) OR (”same sex couple*”) OR (”same sex relation*”) OR (”women loving women”) OR (”men who have sex with men”) OR (”women who have sex with women”) OR (Lesbigay) OR (”Non-Heterosex*”) OR (”Non heterosex*”) OR (GLB) OR (GLBT) OR (GLBTQ) OR (LGB) OR (LGBT) OR (LGBTQ) OR (LGBTQI) OR (LGBTQIA) OR (transgender*) OR (transsexual*) OR (”gender dysphoria”) OR (”gender nonbinary”) OR (”nonbinary gender”) OR (nonbinary) OR (”non binary”) OR (transvesti*) OR (crossgender) OR (”gender change”) OR (”gender transform*”) OR (”gender transition”) OR (genderqueer) OR (”trans female*”) OR (”trans male*”) OR (”trans man”) OR (”trans men”) OR (”trans people”) OR (”trans person”) OR (”trans woman”) OR (”trans women”) OR (”gender identity disorder*”) OR (bicurious) OR (intersex*) OR (Asexual*) OR (”Two-spirit*”) OR (Pansex*) OR (”Gender queer”) OR (Agender*) OR (Bigender*) OR (Pangender*) OR (Omnisexual*) OR (”Gender variant*”) OR (”Gender fluid”)) AND (("Emigrants and Immigrants"[Mesh]) OR (”Emigration and Immigration”[Mesh]) OR (”Refugees”[Mesh]) OR (Refugee*) OR (”War-Related Injuries”[Mesh]) OR (Newcomer*) OR (Settler*) OR (Noncitizen*) OR (Incomer*) OR (Incoming*) OR (Foreign*))

871 hits

CINAHL
((MH "Sexual and Gender Minorities+") OR (queer*) OR (homosexual*) OR (”sexual minorit*”) OR (”sexual orientation*”) OR (”gender minorit*”) OR (lesbian*) OR (gay) OR (bisexual*) OR (”same sex couple*”) OR (”same sex relation*”) OR (”women loving women”) OR (”men who have sex with men”) OR (”women who have sex with women”) OR (Lesbigay) OR (”Non-Heterosex*”) OR (”Non heterosex*”) OR (GLB) OR (GLBT) OR (GLBTQ) OR (LGB) OR (LGBT) OR (LGBTQ) OR (LGBTQI) OR (LGBTQIA) OR (transgender*) OR (transsexual*) OR (”gender dysphoria”) OR (”gender nonbinary”) OR (”nonbinary gender”) OR (nonbinary) OR (”non binary”) OR (transvesti*) OR (crossgender) OR (”gender change”) OR (”gender transform*”) OR (”gender transition”) OR (genderqueer) OR (”trans female*”) OR (”trans male*”) OR (”trans man”) OR (”trans men”) OR (”trans people”) OR (”trans person”) OR (”trans woman”) OR (”trans women”) OR (”gender identity disorder*”) OR (bicurious) OR (intersex*) OR (Asexual*) OR (”Two-spirit*”) OR (Pansex*) OR (”Gender queer”) OR (Agender*) OR (Bigender*) OR (Pangender*) OR (Omnisexual*) OR (”Gender variant*”) OR (”Gender fluid”)) AND ((MH "Emigration and Immigration") OR (MH "Immigrants+") OR (MH "Refugees+") OR (Emigrant*) OR (”War-Related Injur*) OR (”War related Injur*) OR (”War-Related Trauma*”) OR (”War related Trauma*”) OR (Newcomer*) OR (Settler*) OR (Noncitizen*) OR (Incomer*) OR (Incoming*) OR (Foreign*))

582 hits

PsycINFO

((DE "Sexual Minority Groups") OR (DE "Homosexuality") OR (DE "Lesbianism") OR (DE "Male Homosexuality") OR (DE "Intersex") OR (DE "Hermaphroditism") OR (queer*) OR (homosexual*) OR (”sexual minorit*”) OR (”sexual orientation*”) OR (”gender minorit*”) OR (lesbian*) OR (gay) OR (bisexual*) OR (”same sex couple*”) OR (”same sex relation*”) OR (”women loving women”) OR (”men who have sex with men”) OR (”women who have sex with women”) OR (Lesbigay) OR (”Non-Heterosex*”) OR (”Non heterosex*”) OR (GLB) OR (GLBT) OR (GLBTQ) OR (LGB) OR (LGBT) OR (LGBTQ) OR (LGBTQI) OR (LGBTQIA) OR (transgender*) OR (transsexual*) OR (”gender dysphoria”) OR (”gender nonbinary”) OR (”nonbinary gender”) OR (nonbinary) OR (”non binary”) OR (transvesti*) OR (crossgender) OR (”gender change”) OR (”gender transform*”) OR (”gender transition”) OR (genderqueer) OR (”trans female*”) OR (”trans male*”) OR (”trans man”) OR (”trans men”) OR (”trans people”) OR (”trans person”) OR (”trans woman”) OR (”trans women”) OR (”gender identity disorder*”) OR (bicurious) OR (intersex*) OR (Asexual*) OR (”Two-spirit*”) OR (Pansex*) OR (”Gender queer”) OR (Agender*) OR (Bigender*) OR (Pangender*) OR (Omnisexual*) OR (”Gender variant*”) OR (”Gender fluid”)) AND ((DE "Immigration") OR (DE "Human Migration") OR (DE "Refugees") OR (Emigrant*) OR (”War-Related Injur*) OR (”War related Injur*) OR (”War-Related Trauma*”) OR (”War related Trauma*”) OR (Newcomer*) OR (Settler*) OR (Noncitizen*) OR (Incomer*) OR (Incoming*) OR (Foreign*))

1,203 hits

SCOPUS
(("Sexual Minority Group*") OR (queer*) OR (homosexual*) OR (”sexual minorit*”) OR (”sexual orientation*”) OR (”gender minorit*”) OR (lesbian*) OR (gay) OR (bisexual*) OR (”same sex couple*”) OR (”same sex relation*”) OR (”women loving women”) OR (”men who have sex with men”) OR (”women who have sex with women”) OR (Lesbigay) OR (”Non-Heterosex*”) OR (”Non heterosex*”) OR (GLB) OR (GLBT) OR (GLBTQ) OR (LGB) OR (LGBT) OR (LGBTQ) OR (LGBTQI) OR (LGBTQIA) OR (transgender*) OR (transsexual*) OR (”gender dysphoria”) OR (”gender nonbinary”) OR (”nonbinary gender”) OR (nonbinary) OR (”non binary”) OR (transvesti*) OR (crossgender) OR (”gender change”) OR (”gender transform*”) OR (”gender transition”) OR (genderqueer) OR (”trans female*”) OR (”trans male*”) OR (”trans man”) OR (”trans men”) OR (”trans people”) OR (”trans person”) OR (”trans woman”) OR (”trans women”) OR (”gender identity disorder*”) OR (bicurious) OR (intersex*) OR (Asexual*) OR (”Two-spirit*”) OR (Pansex*) OR (”Gender queer”) OR (Agender*) OR (Bigender*) OR (Pangender*) OR (Omnisexual*) OR (”Gender variant*”) OR (”Gender fluid”)) AND (("Immigration") OR ("Human Migration") OR ("Refugees") OR (Emigrant*) OR (”War-Related Injur*) OR (”War related Injur*) OR (”War-Related Trauma*”) OR (”War related Trauma*”) OR (Newcomer*) OR (Settler*) OR (Noncitizen*) OR (Incomer*) OR (Incoming*) OR (Foreign*))

Limits: Article
1,649 hits

Web of Science
(("Sexual Minority Group*") OR (queer*) OR (homosexual*) OR (”sexual minorit*”) OR (”sexual orientation*”) OR (”gender minorit*”) OR (lesbian*) OR (gay) OR (bisexual*) OR (”same sex couple*”) OR (”same sex relation*”) OR (”women loving women”) OR (”men

who have sex with men”) OR (”women who have sex with women”) OR (Lesbigay) OR (”Non-Heterosex*”) OR (”Non heterosex*”) OR (GLB) OR (GLBT) OR (GLBTQ) OR (LGB) OR (LGBT) OR (LGBTQ) OR (LGBTQI) OR (LGBTQIA) OR (transgender*) OR (transsexual*) OR (”gender dysphoria”) OR (”gender nonbinary”) OR (”nonbinary gender”) OR (nonbinary) OR (”non binary”) OR (transvesti*) OR (crossgender) OR (”gender change”) OR (”gender transform*”) OR (”gender transition”) OR (genderqueer) OR (”trans female*”) OR (”trans male*”) OR (”trans man”) OR (”trans men”) OR (”trans people”) OR (”trans person”) OR (”trans woman”) OR (”trans women”) OR (”gender identity disorder*”) OR (bicurious) OR (intersex*) OR (Asexual*) OR (”Two-spirit*”) OR (Pansex*) OR (”Gender queer”) OR (Agender*) OR (Bigender*) OR (Pangender*) OR (Omnisexual*) OR (”Gender variant*”) OR (”Gender fluid”)) AND (("Immigration") OR ("Human Migration") OR ("Refugees") OR (Emigrant*) OR (”War-Related Injur*) OR (”War related Injur*) OR (”War-Related Trauma*”) OR (”War related Trauma*”) OR (Newcomer*) OR (Settler*) OR (Noncitizen*) OR (Incomer*) OR (Incoming*) OR (Foreign*))

1,286 hits

OpenGrey
(("Sexual Minority Group*") OR (queer*) OR (homosexual*) OR (”sexual minorit*”) OR (”sexual orientation*”) OR (”gender minorit*”) OR (lesbian*) OR (gay) OR (bisexual*) OR (”same sex couple*”) OR (”same sex relation*”) OR (”women loving women”) OR (”men who have sex with men”) OR (”women who have sex with women”) OR (Lesbigay) OR (”Non-Heterosex*”) OR (”Non heterosex*”) OR (GLB) OR (GLBT) OR (GLBTQ) OR (LGB) OR (LGBT) OR (LGBTQ) OR (LGBTQI) OR (LGBTQIA) OR (transgender*) OR (transsexual*) OR (”gender dysphoria”) OR (”gender nonbinary”) OR (”nonbinary gender”) OR (nonbinary) OR (”non binary”) OR (transvesti*) OR (crossgender) OR (”gender change”) OR (”gender transform*”) OR (”gender transition”) OR (genderqueer) OR (”trans female*”) OR (”trans male*”) OR (”trans man”) OR (”trans men”) OR (”trans people”) OR (”trans person”) OR (”trans woman”) OR (”trans women”) OR (”gender identity disorder*”) OR (bicurious) OR (intersex*) OR (Asexual*) OR (”Two-spirit*”) OR (Pansex*) OR (”Gender queer”) OR (Agender*) OR (Bigender*) OR (Pangender*) OR (Omnisexual*) OR (”Gender variant*”) OR (”Gender fluid”)) AND (("Immigration") OR ("Human Migration") OR ("Refugees") OR (Emigrant*) OR (”War-Related Injur*) OR (”War related Injur*) OR (”War-Related Trauma*”) OR (”War related Trauma*”) OR (Newcomer*) OR (Settler*) OR (Noncitizen*) OR (Incomer*) OR (Incoming*) OR (Foreign*))

Limits: English
38 hits

Cochrane Library
((MeSH descriptor: [Sexual and Gender Minorities] explode all trees) OR (queer*) OR (homosexual*) OR (”sexual minorit*”) OR (”sexual orientation*”) OR (”gender minorit*”) OR (lesbian*) OR (gay) OR (bisexual*) OR (”same sex couple*”) OR (”same sex relation*”) OR (”women loving women”) OR (”men who have sex with men”) OR (”women who have sex with women”) OR (Lesbigay) OR (”Non-Heterosex*”) OR (”Non heterosex*”) OR (GLB) OR (GLBT) OR (GLBTQ) OR (LGB) OR (LGBT) OR (LGBTQ) OR (LGBTQI) OR (LGBTQIA) OR (transgender*) OR (transsexual*) OR (”gender dysphoria”)

OR (”gender nonbinary”) OR (”nonbinary gender”) OR (nonbinary) OR (”non binary”) OR (transvesti*) OR (crossgender) OR (”gender change”) OR (”gender transform*”) OR (”gender transition”) OR (genderqueer) OR (”trans female*”) OR (”trans male*”) OR (”trans man”) OR (”trans men”) OR (”trans people”) OR (”trans person”) OR (”trans woman”) OR (”trans women”) OR (”gender identity disorder*”) OR (bicurious) OR (intersex*) OR (Asexual*) OR (”Two-spirit*”) OR (Pansex*) OR (”Gender queer”) OR (Agender*) OR (Bigender*) OR (Pangender*) OR (Omnisexual*) OR (”Gender variant*”) OR (”Gender fluid”)) AND ((MeSH descriptor: [Emigrants and Immigrants] explode all trees) OR (MeSH descriptor: [Emigration and Immigration] explode all trees) OR (MeSH descriptor: [Refugees] explode all trees) OR (Refugee*) OR (MeSH descriptor: [War-Related Injuries] explode all trees) OR (Newcomer*) OR (Settler*) OR (Noncitizen*) OR (Incomer*) OR (Incoming*) OR (Foreign*))

Limit: Trials
11 hits
