# Supplementary material for: Post‐migration psychosocial experiences and challenges amongst LGBTQ+ forced migrants: A meta‐synthesis of qualitative reports
Source: J Adv Nurs. 2022 Nov 1;79(1):358–71. doi: 10.1111/jan.15480 (PMC10092230; doi:10.1111/jan.15480)
Supplement: Supplementary file 3 — File 3 [file JAN-79-358-s001.pdf]

Additional File 3. Data extraction tool.

		**Comments**
Study author(s):		
Title of study:		
Journal:		
Date of extraction:		
Date of primary study conducted:		
Year of paper published:		
Study design:		
Method for recruitment:	☐ Consecutive<br>☐ Convenience<br>☐ Purposeful<br>☐ Snowball<br>☐ Not specified<br>Other:	
Data collection method:	☐ Individual interviews<br>☐ Focus groups<br>☐ Written statements/comments/narratives<br>☐ Not specified<br>Other:	
Phenomena of Interest (purpose/aim):	☐ Not specified	

Setting:	☐ Clinical<br>☐ Community<br>☐ Not specified<br>Other:			
Geographical area (country/region):	☐ Not specified			
Participant's characteristics :		n	%	
	**Ethnicity**			
	**Gender**			
	**Sexual orientation**			

Sample size:				
Grant support:	☐ Yes, please specify:<br>☐ No<br>☐ Not specified			
Data analysis:	☐ Content analysis<br>☐ Thematic analysis<br>☐ Not sepcified<br>Other:			
Themes and sub-themes /categories and sub-categories in study findings:	1.<br>2.<br>3.<br>4.<br>5.<br>6.<br>7.<br>8.<br>9.<br>10.			
Author's conclusion:				
Comments:				
