# Supplementary material for: Post‐migration psychosocial experiences and challenges amongst LGBTQ+ forced migrants: A meta‐synthesis of qualitative reports
Source: J Adv Nurs. 2022 Nov 1;79(1):358–71. doi: 10.1111/jan.15480 (PMC10092230; doi:10.1111/jan.15480)
Supplement: Supplementary file 4 — File 4 [file JAN-79-358-s004.pdf]

Additional File 4. Presentation of methodological profile of the included reports.

Report, country	Aim / Research question	Recruitment	Sample: Total sample (n)	Sample: Sexual orientations (n)	Sample: Gender identities[1]	Data collection	Analysis	CASP	JBI
(Akin, 2017), Norway	Focuses on the burden of proof that queer asylum seekers encounter and how they tackle it	Via human rights organization (not specified further)	10	Gay = 8<br>Lesbian = 2	Cisgender men = 8<br>Cisgender women = 2	Interviews	Not specified	4	4
(Alessi et al., 2020), Austria, Netherlands	How do LGBTQ refugees from Islamic societies describe and understand their integration experiences in Austria and the Netherlands	Purposeful	38	Gay = 24<br>Lesbian = 3<br>Bisexual = 3<br>Not specified = 8	Transgender women = 5<br>Q/GNC/NB = 2<br>Transgender men = 1<br>Not specified = 30	Interviews	Thematic analysis	7	9
(Alessi, 2016), USA, Canada	Explore resilience in sexual and gender minority forced migrants	Purposeful	26	Gay = 20<br>Lesbian = 2<br>Not specified = 4	Transgender women = 2<br>Transgender men = 2<br>Not specified = 22	Interviews	Thematic analysis	8	9
(Alessi et al., 2018), Austria, Netherlands	Investigate how traumatic stress shaped the migration experiences of LGBTQ refugees	Purposeful, snowball	38	Gay = 24<br>Lesbian = 3<br>Bisexual = 3<br>Not specified = 8	Transgender women = 5<br>Q/GNC/NB = 2<br>Transgender men = 1<br>Not specified = 30	Interviews	Grounded theory/constant comparative analysis	8	9
(Alessi et al., 2021), Austria, Netherlands	Understand how LGBTQ refugees who fled from Islamic societies experienced their religious identities and how those religious identities changed and evolved	Purposeful, snowball	34	Gay = 20<br>Lesbian = 3<br>Bisexual = 3<br>Not specified = 8	Transgender women = 5<br>Q/GNC/NB = 2<br>Transgender men = 1<br>Not specified = 26	Interviews	Thematic	9	10
(Cerezo et al., 2014), USA	Add to the paucity of research on LGBT immigrants in the mental health disciplines. Understand what motivated women to migrate as well as their negotiation of multiple culturally marked identities in key institutions, like the workforce and health care settings.	Convenience	10	Heterosexual = 9<br>Bisexual = 1	Transgender women = 10	Interviews	Case study/ Case analysis	9	10
(Dhoest, 2020), Belgium	Better understand the uses and limitations of social media for LGBTQ refugees, avoiding the dual trap of overly euphoric or dysphoric accounts	Not specified	9	Gay = 9	Cisgender men = 9	Interviews	Not specified	4	3
(Dhoest, 2019), Belgium	Explore the telling, and assessing, of narratives about sexual identity within the asylum procedure; Address the actual role of the procedure, by analysing the narratives of people in different stages of the procedure	Convenience	8	Gay = 8	Cisgender men = 8	Interviews	Not specified	5	8
(Golembe et al., 2020),	Investigated LGBTQ* refugees' experiences of distal and proximal minority stressors as well as	Convenience	26	Gay = 18<br>Bisexual = 3	Cisgender men = 21<br>Transgender women = 3	Focus groups	Thematic	9	10

Germany	their mental health burdens during the post-migration period			Not specified = 5	Q/GNC/NB = 2				
(Held, 2022), Germany, Italy, United Kingdom	Explores LGBTIQ+ refugees' experiences in different spaces such as LGBTIQ+ support groups and night-time leisure spaces, as well as intimate relationships	Snowball	Not specified	Not specified	Not specified	Interviews, Focus groups	Not specified	7	7
(Karimi, 2020b), Canada	Focus on the case of gay refugees and analyze their pre-migration transnational lives and understandings of the asylum process, their post-migration transnational ties, and their activism practices	Snowball	19	Gay = 19	Cisgender men = 19	Interviews	Thematic	6	5
(Karimi, 2020a), Canada	Connect the empirical and theoretical debates around refugee integration and argue that over-reliance on refugees' deployment of social capital for integration has grave shortcomings for their senses of belonging	Snowball	19	Gay = 19	Cisgender men = 19	Interviews	Thematic ethnographic analysis	6	6
(Karimi, 2021), Canada	Explore the ways that past experiences and histories of socialization inform integration beyond the limits of ethnicity and at the intersections of sexuality, gender, race, and religion; underline the importance of sexuality for understanding intra- and inter-group diversities and to argue for the insufficiency of the ethnic lens in studying migration and integration	Snowball	19	Gay = 19	Cisgender men = 19	Interviews and observations	Thematic ethnographic analysis	7	7
(Kahn, 2015a), USA	Does hailing from a Muslim society afford productive social connections with coethnic others; What are the social costs of being gender role outlaws; How do those costs or benefits express themselves throughout the asylum-seeking experience	Purposeful	7	Gay = 7	Cisgender men = 7	Interviews	Grounded theory/constant comparative analysis	8	8
(Kahn et al., 2018), Canada	Understand the facilitators of and barriers to mental health care for LGBT forced migrants in Canada	Purposeful	7	Gay = 4<br>Lesbian = 2<br>Not specified = 1	Transgender = 1<br>Not specified = 6	Interviews	Thematic analysis	8	9
(Kahn & Alessi, 2018), Canada	How do service providers and LGBT forced migrants describe the psychological dimensions of claiming refugee status on the basis of SOGI in Canada; To what extent do LGBT forced migrants experience psychological consequences related to pursuing a refugee claim in Canada, and to what extent do such consequences persist after the claim is completed	Purposeful, snowball	7	Gay = 4<br>Lesbian = 2<br>Not specified = 1	Transgender = 1<br>Not specified = 6	Interviews	Grounded theory/constant comparative analysis	9	10

(Kahn, 2015b), USA	Elucidate relationships between Muslim asylum seekers and Islamic faith; Whether, and how, was faith connected to expectations for gender role conformity in their societies of origin; What were their experiences of reconciling faith with their gender role non-conformity; How did they subsequently experience relationships with God post asylum; How did social workers or other mental health and social service providers assist or facilitate explorations of religious faith and affiliation	Purposeful	7	Gay = 7	Cisgender men = 7	Interviews	Grounded theory/constant comparative analysis	7	7
(Kostenius et al., 2021), Sweden	Explore the lived experiences of LGBTQ migrants participating in a civil society group during their migration process and their reflections about the future	Purposeful	11	Gay = 9<br>Lesbian = 1<br>Bisexual = 1	Cisgender men = 7<br>Cisgender women = 2<br>Transgender = 2	Interviews	Interpretative phenomenological analysis	9	10
(Lee & Brotman, 2011), Canada	Through critical analysis of the interrelated themes of identity, refugeeness, and belonging we hope to contribute to queer migration scholarship in North America; Investigate the ways in which refugee policies, social institutions, and dominant discourses contribute to the sociopolitical construction of sexual minority refugees; Centering the experiences of sexual minority refugees themselves will help foster an understanding of how they respond to and resist constraining sociocultural forces; Identify the tensions between the discursive complicities and material consequences of entering into sexual rights based discourses in order to promote sexual minority refugee rights and conclude with an exploration of strategies for increasing protection of and advocacy with sexual minority refugees	Snowball	24	Not specified = 24	Cisgender men = 11<br>Cisgender women = 8<br>Transgender women = 5	Interviews	Thematic; Grounded theory/constant comparative analysis	7	8
(Llewellyn, 2021), USA	Understand the lived experience of LGBTQ asylum applicants	Convenience, snowball	18	Gay = 14<br>Lesbian = 3<br>Not specified = 1	Cisgender men = 14<br>Cisgender women = 3<br>Transgender women = 1	Interviews	Thematic analysis	8	9
(Logie et al., 2016), Canada	Explore experiences of social support group participation among LGBT newcomers and refugees in an urban city	Convenience, snowball	29	Bisexual = 12<br>Gay = 9<br>Lesbian = 5<br>Heterosexual = 1<br>Other = 1<br>Not specified = 1	Cisgender men = 15<br>Cisgender women = 11<br>Transgender = 3	Focus groups	Narrative thematic analysis	8	8
(Mulé, 2021),	Examine the mental health issue and needs of	Convenience	92	Gay = 38	Cisgender men = 52	Focus groups	Thematic;	7	7

Canada	LGBTQ asylum seekers and refugees from a critical psychology perspective; Critically understand how LGBTQ asylum seekers and refugees are impacted by their experience via the following research questions: Did they have adequate access to information and resources; How did they manage socio-cultural shifts in understanding the concept of identities; How did they navigate the refugee claims process; How did they manage trauma; How has their experience impacted their mental health			Bisexual = 24<br>Lesbian = 18<br>Heterosexual = 2<br>Queer = 2<br>Pansexual = 1<br>Not specified = 7	Cisgender women = 33<br>Q/GNC/NB = 7<br>Transgender = 2<br>Two-spirit = 2		Participatory action research		
(Murray, 2014a), Canada	Provide examples of ''real versus fake'' refugee talk among refugee claimants and their support workers that elucidate the centrality of homonationalism as an underlying precept of the refugee determination system and neoliberal, multiculturalist discourses	Not specified	54	Not specified = 54	Not specified = 54	Interviews	Not specified	4	1
(Murray, 2014b), Canada	Explores diverse discourses, experiences, and meanings of home for SOGI refugees	Not specified	54	Not specified = 54	Not specified = 54	Interviews	Not specified	3	2
(Novitskaya, 2021), USA	Discusses the effects of Russian state-sponsored homophobia and sexual citizenship, and it is based on empirical data collected during a transnational ethnographic study of the Russian-speaking LGBTQI+ community	Convenience, snowball	Not specified	Gay = 12<br>Lesbian = 8<br>Bisexual = 3<br>Queer = 2<br>Pansexual = 1	Cisgender men = 15<br>Cisgender women = 12<br>Bigender = 1<br>Intersex = 1	Interviews, observations	Ethnographic analysis	6	4
(Oren & Gorshkov, 2021), USA	Explore and interpret lived experience of the Russian-speaking LGBT+ immigrants in the United States	Purposeful, snowball	9	Not specified = 9	Not specified = 9	Interviews	Interpretative phenomenological analysis	9	10
(Rosati et al., 2021), Italy	Enriching the empirical literature and providing a useful instrument to gather information on the topic of the forced migration of the transgender population; To collect preliminary data on transgender refugees' experiences of pre- and post-migration and their intersecting ethnic and gender identities, using a semi-structured interview grounded in minority stress and intersectionality perspectives; Investigating (1) experiences related to the pre- and post-migratory journey, (2) the intersectionality of multiple minority identities, such as ethnic and gender identities, and (3) risk and protective factors related to migration and intersectionality	Not specified	5	Not specified = 5	Trangender woman = 4<br>Non-binary transgender intersex = 1	Interviews	Thematic	8	10
(Wimark,	Add to the debate on liminality in migration	Convenience,	22	Men who desire	Cisgender men = 22	Interviews	Grounded	8	8

2019), Sweden	studies by considering a multitude of spaces, rather than simply state-controlled spaces, as liminal to refugees; Contribute to the understanding of liminality by suggesting the possibility of perpetual liminality after gaining asylum	snowball		men = 22			theory/constant comparative analysis		
(Wimark, 2021), Sweden	Contribute to the latter stream of literature by considering the ways in which the governance of asylum accommodation affects queer asylum seekers; Map the governance of the Swedish refugee accommodation system and analyse how it affects queer asylum seekers	Convenience, snowball	34	Not specified = 34	Not specified = 34	Interviews	Not specified	7	7

[1] If presented as man or woman without further specification (e.g. "gay man" or "lesbian woman"), we have surmised that these participants were cisgender

References

Akin, D. (2017). Queer asylum seekers: Translating sexuality in Norway. *Journal of Ethnic and Migration Studies*, *43*(3), 458–474. https://doi.org/10.1080/1369183X.2016.1243050

Alessi, E. J. (2016). Resilience in sexual and gender minority forced migrants: A qualitative exploration. *Traumatology*, *22*(3), 203–213. psyh. https://doi.org/10.1037/trm0000077

Alessi, E. J., Greenfield, B., Kahn, S., & Woolner, L. (2021). (Ir)reconcilable identities: Stories of religion and faith for sexual and gender minority refugees who fled from the Middle East, North Africa, and Asia to the European Union. *Psychology of Religion and Spirituality*, *13*(2), 175–183. psyh. https://doi.org/10.1037/rel0000281

Alessi, E. J., Kahn, S., Greenfield, B., Woolner, L., & Manning, D. (2020). A qualitative exploration of the integration experiences of LGBTQ refugees who fled from the Middle East, North Africa, and Central and South Asia to Austria and the Netherlands. *Sexuality Research & Social Policy: A Journal of the NSRC*, *17*(1), 13–26. psyh. https://doi.org/10.1007/s13178-018-0364-7

Alessi, E. J., Kahn, S., Woolner, L., & Van Der Horn, R. (2018). Traumatic Stress Among Sexual and Gender Minority Refugees From the Middle East, North Africa, and Asia Who Fled to the European Union. *Journal of Traumatic Stress*, *31*(6), 805–815. https://doi.org/10.1002/jts.22346

Cerezo, A., Morales, A., Quintero, D., & Rothman, S. (2014). Trans migrations: Exploring life at the intersection of transgender identity and immigration. *Psychology of Sexual Orientation and Gender Diversity*, *1*(2), 170–180. psyh. https://doi.org/10.1037/sgd0000031

Dhoest, A. (2019). Learning to be gay: LGBTQ forced migrant identities and narratives in Belgium. *Journal of Ethnic and Migration Studies*, *45*(7), 1075–1089. Scopus. https://doi.org/10.1080/1369183X.2017.1420466

Dhoest, A. (2020). Digital (dis)connectivity in fraught contexts: The case of gay refugees in Belgium. *European Journal of Cultural Studies*, *23*(5), 784–800. Scopus. https://doi.org/10.1177/1367549419869348

Golembe, J., Leyendecker, B., Maalej, N., Gundlach, A., & Busch, J. (2020). Experiences of Minority Stress and Mental Health Burdens of Newly Arrived LGBTQ* Refugees in Germany. *Sexuality Research and Social Policy*. https://doi.org/10.1007/s13178-020-00508-z

Held, N. (2022). “As queer refugees, we are out of category, we do not belong to one, or the other”: LGBTIQ+ refugees’ experiences in “ambivalent” queer spaces. *Ethnic and Racial Studies*, 1–21. https://doi.org/10.1080/01419870.2022.2032246

Kahn, S. (2015a). Cast out: “Gender Role Outlaws” seeking asylum in the west and the quest for social connections. *Journal of Immigrant and Refugee Studies*, *13*(1), 58–79. Scopus. https://doi.org/10.1080/15562948.2014.894169

Kahn, S. (2015b). Experiences of Faith for Gender Role Non-Conforming Muslims in Resettlement: Preliminary Considerations for Social Work Practitioners. *British Journal of Social Work*, *45*(7), 2038–2055. cin20. https://doi.org/10.1093/bjsw/bcu060

Kahn, S., & Alessi, E. J. (2018). Coming out under the gun: Exploring the psychological dimensions of seeking refugee status for lgbt claimants in Canada. *Journal of Refugee Studies*, *31*(1), 22–41. Scopus. https://doi.org/10.1093/jrs/fex019

Kahn, S., Alessi, E. J., Kim, H., Woolner, L., & Olivieri, C. J. (2018). Facilitating mental health support for LGBT forced migrants: A qualitative inquiry. *Journal of Counseling & Development*, *96*(3), 316–326. psyh. https://doi.org/10.1002/jcad.12205

Karimi, A. (2020a). Limits of Social Capital for Refugee Integration: The Case of Gay Iranian Male Refugees’ Integration in Canada. *International Migration*, *58*(5), 87–102. Scopus. https://doi.org/10.1111/imig.12691

Karimi, A. (2020b). Refugees’ Transnational Practices: Gay Iranian Men Navigating Refugee Status and Cross-border Ties in Canada. *Social Currents*, *7*(1), 71–86. Scopus. https://doi.org/10.1177/2329496519875484

Karimi, A. (2021). Sexuality and integration: A case of gay Iranian refugees’ collective memories and integration practices in Canada. *Ethnic and Racial Studies*, *44*(15), 2857–2875. https://doi.org/10.1080/01419870.2018.1550207

Kostenius, C., Hertting, K., Pelters, P., & Lindgren, E.-C. (2021). From Hell to Heaven? Lived experiences of LGBTQ migrants in relation to health and their reflections on the future. *Culture, Health & Sexuality*, 1–13. https://doi.org/10.1080/13691058.2021.1983020

Lee, E. O. J., & Brotman, S. (2011). Identity, refugeeness, belonging: Experiences of sexual minority refugees in Canada. *Canadian Review of Sociology = Revue Canadienne de Sociologie*, *48*(3), 241–274. https://doi.org/10.1111/j.1755-618x.2011.01265.x

Llewellyn, C. (2021). Captive While Waiting to Be Free: Legal Violence and LGBTQ Asylum Applicant Experiences in the USA. *Sexuality Research and Social Policy*, *18*(1), 202–212. Scopus. https://doi.org/10.1007/s13178-020-00449-7

Logie, C. H., Lacombe-Duncan, A., Lee-Foon, N., Ryan, S., & Ramsay, H. (2016). “It’s for us -newcomers, LGBTQ persons, and HIV-positive persons. You feel free to be”: A qualitative study exploring social support group participation among African and Caribbean lesbian, gay, bisexual and transgender newcomers and refugees in Toronto, Canada. *BMC International Health and Human Rights*, *16*(1), 18. https://doi.org/10.1186/s12914-016-0092-0

Mulé, N. J. (2021). Mental health issues and needs of LGBTQ+ asylum seekers, refugee claimants and refugees in Toronto, Canada. *Psychology and Sexuality*. Scopus. https://doi.org/10.1080/19419899.2021.1913443

Murray, D. A. B. (2014a). Real queer: “Authentic” LGBT refugee claimants and homonationalism in the Canadian Refugee System. *Anthropologica*, *56*(1), 21–32. psyh.

Murray, D. A. B. (2014b). The challenge of home for sexual orientation and gendered identity refugees in Toronto. *Journal of Canadian Studies*, *48*(1), 132–152. Scopus. https://doi.org/10.1353/jcs.2014.0019

Novitskaya, A. (2021). Sexual Citizens in Exile: State-Sponsored Homophobia and Post-Soviet LGBTQI+ Migration. *Russian Review*, *80*(1), 56–76. Scopus. https://doi.org/10.1111/russ.12298

Oren, T., & Gorshkov, A. (2021). Lived Experiences of Recent Russian-Speaking LGBT+ Immigrants in the United States: An Interpretive Phenomenological Analysis. *Journal of LGBT Issues in Counseling*, *15*(3), 290–309. cin20. https://doi.org/10.1080/15538605.2021.1914278

Rosati, F., Coletta, V., Pistella, J., Scandurra, C., Laghi, F., & Baiocco, R. (2021). Experiences of Life and Intersectionality of Transgender Refugees Living in Italy: A Qualitative Approach. *International Journal of Environmental Research and Public Health*, *18*(23). https://doi.org/10.3390/ijerph182312385

Wimark, T. (2019). Homemaking and perpetual liminality among queer refugees. *Social and Cultural Geography*. Scopus. https://doi.org/10.1080/14649365.2019.1619818

Wimark, T. (2021). Housing policy with violent outcomes–the domestication of queer asylum seekers in a heteronormative society. *Journal of Ethnic and Migration Studies*, *47*(3), 703–722. Scopus. https://doi.org/10.1080/1369183X.2020.1756760
