# Supplementary material for: Post‐migration psychosocial experiences and challenges amongst LGBTQ+ forced migrants: A meta‐synthesis of qualitative reports
Source: J Adv Nurs. 2022 Nov 1;79(1):358–71. doi: 10.1111/jan.15480 (PMC10092230; doi:10.1111/jan.15480)
Supplement: Supplementary file 5 — File 5 [file JAN-79-358-s003.pdf]

Additional File 5. Summary of the synthesis in the theme Experiencing psychological distress and encountering difficult challenges while trying to find a way to survive in host country, with frequency effect sizes (FES).

CATEGORY, SUB-CATEGORY AND CONTENT	ILLUSTRATIVE QUOTE	FES
**Category: Intersectional challenges encountered as an LGBTQ+ forced migrant** (Akin, 2017; Alessi, 2016; Alessi et al., 2018, 2020, 2021; Cerezo et al., 2014; Dhoest, 2019, 2020; Golembe et al., 2020; Held, 2022; Kahn, 2015a, 2015b; Kahn & Alessi, 2018; Karimi, 2020b, 2020a, 2021; Kostenius et al., 2021; Lee & Brotman, 2011; Llewellyn, 2021; Logie et al., 2016; Mulé, 2021; Murray, 2014a, 2014b; Novitskaya, 2021; Oren & Gorshkov, 2021; Rosati et al., 2021; Wimark, 2019, 2021)		**97 %**
Sub-category: Encountering intersectional discrimination, prejudice, and violence (Akin, 2017; Alessi, 2016; Alessi et al., 2018, 2020, 2021; Cerezo et al., 2014; Dhoest, 2019; Golembe et al., 2020; Held, 2022; Kahn, 2015a, 2015b; Karimi, 2020b, 2020a, 2021; Kostenius et al., 2021; Lee & Brotman, 2011; Llewellyn, 2021; Murray, 2014a; Novitskaya, 2021; Oren & Gorshkov, 2021; Rosati et al., 2021; Wimark, 2019, 2021)		79 %
A range of perpetrators exposed migrants to discrimination and violence, including:		
Diaspora coethnic community members, compatriots and/or other forced migrants (Akin, 2017; Alessi et al., 2018, 2020; Cerezo et al., 2014; Dhoest, 2019; Golembe et al., 2020; Kahn, 2015a; Karimi, 2020b, 2020a; Kostenius et al., 2021; Lee & Brotman, 2011; Llewellyn, 2021; Oren & Gorshkov, 2021; Wimark, 2019, 2021)	*However, once these participants attempted to integrate into the host society, they were surprised to find that they continued to encounter homophobia and transphobia from members of their diaspora communities. For example, a transgender female in Amsterdam, Janie, reported that she experienced transphobic discrimination by one of her compatriots who was working in a local government office. Once Janie left the office, she explained that she felt so hurt that she began crying: "I cannot forget that day. That she disrespect me a lot."* (Alessi et al., 2020)	52 %
Mainstream community members (Alessi et al., 2020; Dhoest, 2019; Golembe et al., 2020; Held, 2022; Karimi, 2020a; Kostenius et al., 2021; Llewellyn, 2021; Murray, 2014a; Wimark, 2019)	*In addition to being targeted by other refugees, participants' intersecting identities also placed them at risk of prejudice and discrimination from mainstream community members as they tried to integrate into the host society. However, there were differences in how participants spoke about this type of discrimination. Those in Vienna expressed that it manifested in subtle and overt ways, while those in Amsterdam mostly described it as subtle.* (Alessi et al., 2020)	31 %
LGBTQ+ community members (Alessi et al., 2020; Golembe et al., 2020; Held, 2022; Kahn, 2015a; Karimi, 2021; Lee & Brotman, 2011; Llewellyn, 2021; Oren & Gorshkov, 2021)	*When LGBTIQ+ claimants are able to enter queer spaces, they often encounter racism. Alphaeus (Germany) said that in gay clubs "sometimes you may find some people who discriminate you, but they will discriminate you because of your colour, not [for] being a gay". He told us of an experience where he and his friends were "bullied" by "fellow gay people" in a gay club in Munich, who "are bullying because you are Black" and questioned "why are you here?"* (Held, 2022)	28 %
Official workers (Alessi et al., 2018; Cerezo et al., 2014; Held, 2022; Lee & Brotman, 2011; Murray, 2014a; Rosati et al., 2021)	*Several women shared stories of violence that in- cluded verbal, physical, and sexual assault from family members, community members, and human smugglers while en route to the United States, and authority figures (policemen) both in their country of origin and the United States.*(Cerezo et al., 2014)	21 %
People at shelters (Held, 2022; Lee & Brotman, 2011; Wimark, 2021)	*Interviews with the queer asylum seekers also provided examples of SMA placement staff or accommodation staff asking them to hide and blend in (and Interview, E), which is contradictory to the way they are expected to behave in the asylum process.* (Wimark, 2021)	10 %
Employers (Lee & Brotman, 2011; Llewellyn, 2021)	*Many of the trans refugees described the profound impact of transphobia in blocking their employment opportunities, due to having a masculine name while presently as feminine.* (Lee & Brotman, 2011)	7 %
Health professionals (Golembe et al., 2020; Rosati et al., 2021)	*A trans* participant described a speci c situation when they were seeking treatment at a hospital and felt discriminated against for being both trans* and not able to speak German. I was in the hospital ... and they refused to talk to me because I don't speak German. The doctors did not talk to me. Maybe they get confused because my gender in my documents and my personality isn't the same. There it shows me as a man, but I am a female.* (Golembe et al., 2020)	7 %

Trusted loved ones and family members (Cerezo et al., 2014; Wimark, 2019)	*For Hamza, for example, ostracism only happened in later life when his mother, who had fled to Sweden and gained asylum, fell ill, and he moved there to care for her: Hamza (45): "Before I am about to leave, he [my brother] comes in to my room. I am sleeping with him [a man] in the bed. I have fun with him, sex with him, we were naked of course. My brother, strong brother, just opens the door, like, boom, and they see me, and they push me, like, boom, bye, you´re an idiot; you are very very bad. He swings at me, and I get my stuff and go around him. After, I go back to my family. He said to me that he would call [person in country], and if you go back to [country], he will kill you".* (Wimark, 2019)	7 %
The municipality (Rosati et al., 2021)	*Ayoub reported two episodes of discrimination: one at their local Provincial Police Bureau and the other at a Provincial Centre for Adult Education: "So I've experienced it a lot in the institutional office–let's say official–in the police station, in the hospital, in the school, in the municipality, in the post office, everywhere–in the revenue agency, of course."* (Rosati et al., 2021)	7 %
The post office (Rosati et al., 2021)		
Schools (Rosati et al., 2021)		
Religious doctrines and religious leaders (Kahn, 2015b)	*Although religion, faith and/or spirituality factored into participants' lives in various ways, a persistent theme across interview transcripts was the experience of religion or religious leaders as enforcing gender role conformity and/ or vilifying gender role non-conformity.* (Kahn, 2015b)	7 %
Subtle and overt forms of discrimination, marginalization, and social exclusion related to racism, trans phobia, and homophobia were experienced; often based on their intersecting identities, such as ethnicity, sexual orientation, and gender identity (Alessi et al., 2020, 2021; Cerezo et al., 2014; Golembe et al., 2020; Held, 2022; Karimi, 2020a, 2021; Lee & Brotman, 2011; Llewellyn, 2021; Murray, 2014a; Oren & Gorshkov, 2021; Rosati et al., 2021).	*Participants reported discrimination experiences based on numerous and intersecting attributes. Specifically, participants explained that they are discriminated against based on their gender identity/sexual orientation, their ethnic background, as well as on their status as refugees.* (Golembe et al., 2020)	41 %
Migrants were targets of repeated intense violence, threats, and traumatic events, involving serious danger and distress (Cerezo et al., 2014; Lee & Brotman, 2011; Llewellyn, 2021; Novitskaya, 2021; Wimark, 2019, 2021)	*The women in our sample shared numerous stories of violence and trauma that highlighted the serious dangers faced by many transgender immigrant women. The hardships participants encountered involved socioemotional rejection from key interpersonal relations as well as targeted violence from trusted loved ones, community members, and authority figures, like the police. These intense, regular experiences contributed to psychological distress for several of the participants.* (Cerezo et al., 2014)	21 %
Exploitation and exotification was also experienced, including being expected to perform sexual acts in return for favors (Held, 2022; Karimi, 2021; Wimark, 2021)	*Gay Iranian men are generally sexualized, racialized, and margina- lized in Canadian gay communities. One participant said: "I feel like they [Canadians] want me, sorry, but only for a one-night stand because I'm darker, I know they say I'm exotic". He continued, "I feel wanted, for sex, but not for anything more. They have their own friends and communities, and I do not think I have any place there".* (Karimi, 2021)	10 %
Illustrating the breadth and impact of these discrimination, prejudice, and violence, migrants described daily experiences of discrimination and that they encountered it everywhere (Golembe et al., 2020; Lee & Brotman, 2011; Oren & Gorshkov, 2021)	*Additionally, discrimination was described by several participants to occur daily, from several o ender groups, such as other refugees, white Germans and within the LGBTQ* community. Overall, participants reported feeling as if dis- crimination could happen anywhere and by anyone.* (Golembe et al., 2020)	10 %
Migrants experienced social exclusion, alienation, and marginalization in communities and public spaces, as these were often geared towards well-established homonormative, cis-gendered, and/or white native persons (Held, 2022; Karimi, 2020a, 2021)	*For Amis (focus group, Germany), these exclusionary practices went so far that he was only let into gay bars when he was with a white person: "For me, I've gone to several bars because I'm a proud gay, many people know me and my partner in Munich, a lot of people, and know I'm gay. I've gone to many bars and have a profile, like I even know the bouncers. They tell me "go!", unless when I'm with a white man or a white friend, they let me enter. But when I'm alone ... but they know me of course. So I've seen it and I have proof."* (Held, 2022)	10 %

Some family members tried to change their LGBTQ+ identity (Golembe et al., 2020; Wimark, 2019)	*For instance, one participant explained that they stayed with relatives who wanted to change their sexual orientation. In consequence, they tried to hide their identity from their family as much as possible: "The place I stayed at with my family was small. And my family was around me all the time. And as I said, I had to stop living the gay life. I truly became straight. I seemed straight. I was only able to meet up with people if I traveled to another city."* (Golembe et al., 2020)	7 %
Some experienced worse discrimination than before migrating (Golembe et al., 2020)	*While all participants reported experiences of discrimination, a subgroup of participants seemed to be especially a ected. For those, there was a persistent theme that their experiences of victimization and discrimination in Germany were worse than before ight. Especially regarding the frequency of experienced discrimination, they reported a strong increase.* (Golembe et al., 2020)	3 %
Sub-category: Stressful circumstances (Akin, 2017; Alessi, 2016; Alessi et al., 2020; Dhoest, 2020; Golembe et al., 2020; Held, 2022; Kahn, 2015a; Kahn & Alessi, 2018; Karimi, 2020a; Kostenius et al., 2021; Lee & Brotman, 2011; Llewellyn, 2021; Logie et al., 2016; Mulé, 2021; Murray, 2014b; Novitskaya, 2021; Oren & Gorshkov, 2021; Wimark, 2019, 2021)		66 %
Asylum claiming process represented a considerable challenge for themselves and others, when tasked with having to collect evidence, repeatedly retell traumatic experiences, and describe intimate details during the hearings, which was further compounded when experiencing disbelief among caseworkers and having to conform to stereotypes (Akin, 2017; Alessi, 2016; Alessi et al., 2020; Dhoest, 2020; Held, 2022; Kahn, 2015a; Kahn & Alessi, 2018; Karimi, 2020a; Kostenius et al., 2021; Lee & Brotman, 2011; Llewellyn, 2021; Logie et al., 2016; Mulé, 2021; Murray, 2014b; Novitskaya, 2021; Oren & Gorshkov, 2021; Wimark, 2019, 2021)	*Forced migrants expressed how difficult it was for SOGI claimants to recall their history of persecution, which usually extended back to childhood. Jimmy, a refugee who identified as gay, described how the claims process caused him to retrieve painful memories: "They start to pick on the aspect of fear; of course, that's the basis of your claim. They interrogate you: 'Why are you afraid in your country?' It takes you back emotionally to childhood and adulthood. Coming into this, you would have already put a lot of this behind you. Your lawyer encourages you to go even deeper and, at the hearing, even deeper: You've already opened a Pandora's box; it's hard to close it. I started to see a psychologist. It brought a lot of things up, and they stay with you. And, typically, I'm a strong a person."* (Kahn & Alessi, 2018)	62 %
Long and unpredictable asylum processes involved having to stay in a state of precariousness, uncertainty, and frustration (Alessi, 2016; Alessi et al., 2020; Held, 2022; Lee & Brotman, 2011; Llewellyn, 2021; Murray, 2014b; Novitskaya, 2021; Oren & Gorshkov, 2021)	*Eight participants expressed frustration about a long and unpredictable asylum process: [Sasha] "We have no guarantees. It's hard to imagine how much stress this is. Those who have not experienced this will not know; those who have not come here do not yet know that this is terrible stress."* (Oren & Gorshkov, 2021)	28 %
Living and interacting with other refugees with similar ideals as in their countries of origin, most prominently in refugee centers, was a stressful situation for many LGBTQ+ migrants (Akin, 2017; Held, 2022; Karimi, 2020a; Kostenius et al., 2021; Wimark, 2019, 2021)	*Channels for appropriating a visible and political queer identity are not easily accessible to all asylum seekers, for various reasons. Jess is a lesbian asylum seeker who lives in one of the remote asylum reception centres in Norway, and therefore does not have the opportunity to commute to big cities in Norway where the majority of gay culture is concentrated. She mentions feeling very lonely and threatened among other asylum seekers in the reception centre, who, according to her, make it impossible for her to speak about and act on her sexual orientation.* (Akin, 2017)	21 %
Experiencing ruptures in relationships or having to relinquish relationships involved persistent grief, ambivalence, and missing their previous homes or their family members (Held, 2022; Kahn, 2015a; Karimi, 2020a; Mulé, 2021)	*Four male respondents were faced with complete ruptures—or fear of ruptures—in family bonds because of their homosexuality: "You know you sit there with your sisters, and feel guilty, you know, because my lifestyle is different. So it's very hard for me to communicate with my relatives, with my own brothers … It's just like a completely cut-off, between, between us."* (Kahn, 2015a)	14 %
Those experiencing rapid asylum processes felt pressured to talk when not feeling ready to do so (Alessi, 2016; Kahn, 2015a; Llewellyn, 2021)	*Under the new system, they are expected to tell their stories quickly and co- herently, but they are not always ready to recount their perse- cution or to disclose their sexual orientation. John, a gay man from an African country, referred specifically to the "quick process" and its impact on his mental health: "With a quick process, I believe coming into the country and still having the burden of all that you have had to do to protect yourself, to save yourself in your country of origin, um to be able to go to an interview very quickly could be a disadvantage to some people because they have not been able to tell their story long enough to feel comfortable. I can see someone walking*	10 %

	into an inter- view and still holding back because they are still having that mindset of being back in the country and not being able to fully express their thoughts on certain issues. I find that even with myself. I am still trying to navigate. Who can I really be honest with? Who can I share my true self? I faced so much rejection trying to be real and authentic and transparent in my country so that is a challenge in and of itself." (Llewellyn, 2021)	
Living unemployed, searching for jobs, and having to take jobs with poor workplace cultures was a stressful and challenging process (Karimi, 2020a; Oren & Gorshkov, 2021)	*All nine participants highlighted the stresses and challenges of the job search. While finding survival jobs without speaking English in the generally intolerant ethnic communities was hard, later, the feelings of frustration developed over the entry-level and low-paying jobs.* (Oren & Gorshkov, 2021)	7 %
Upon arrival, migrants experienced psychological distress when placed in detention (Alessi, 2016; Lee & Brotman, 2011)	*The latter quote describes the experience of a gay man and his partner who were detained at a detention center for 10 days because of being iden- tified as a ''Flight Risk'' by Canadian government authorities. Upon arrival at the detention center, the participant was physically separated from his partner, even though a government official had previously told him that this would not happen. Because they were living on separate floors, they could only see each other once a day (during a 30 minute break). "the worst part was that we didn't know what's gonna be the next step, what's gonna happen to us and we couldn't really communicate with each other... there was this unnecessary excessive excitement and anxiety whenever I would see him... I wanted to cry but I couldn't... we wanted to hug and kiss, but then it is a detention center and there are so many people out there."* (Lee & Brotman, 2011)	7 %
Needing to wait a long time for work permits impacted psychological well-being negatively (Llewellyn, 2021)	*The theme of prolonged uncertainty makes explicit the ways in which extended asylum hearing timelines and delayed work authorization impacts the psychological well-being of LGBTQ applicants.* (Llewellyn, 2021)	3 %
Living in urban areas was related to discomfort and stress (Wimark, 2021)	*"I lived in my apartment, I lived alone, but now I am living with people. It is very hard if you want to meet someone. [...] And it is a lot of stress here. Every day, running for the bus, running for the subway. And that is very hard for me. I liked [rural town] much better than Stockholm."* (Wimark, 2021)	3 %
Sub-category: Coping with and countering discrimination and stress (Alessi et al., 2018; Cerezo et al., 2014; Dhoest, 2019, 2020; Golembe et al., 2020; Held, 2022; Kahn, 2015a; Karimi, 2020b, 2020a, 2021; Lee & Brotman, 2011; Llewellyn, 2021; Mulé, 2021; Murray, 2014a, 2014b; Oren & Gorshkov, 2021; Wimark, 2019, 2021)		62 %
Migrants tried to cope and counter discrimination by concealing their LGBTQ+ identity or taking distance from the LGBTQ+ scene; the need to conceal their LGBTQ+ identity was most prominent being when living in refugee camps or interacting with compatriots (Alessi et al., 2018; Cerezo et al., 2014; Dhoest, 2019, 2020; Golembe et al., 2020; Held, 2022; Kahn, 2015a; Karimi, 2020b, 2020a, 2021; Lee & Brotman, 2011; Mulé, 2021; Murray, 2014a, 2014b; Oren & Gorshkov, 2021; Wimark, 2019, 2021)	*In fact, many of our participants went back "into the closet" out of the fear of experiencing homophobic and/or transphobic violence in the asylum accommodation centres in which they were living and which are, like most spaces in society, heteronormative and cis-gendered spaces.* (Held, 2022)	59 %
Migrants expressed few or no ways to counter discrimination and violence (Cerezo et al., 2014; Golembe et al., 2020; Llewellyn, 2021; Wimark, 2021)	*The asylum seekers themselves confirmed in the interviews that they had been given the advice to contact the police if harassment occurred or to get in touch with the staff about homo- and transphobia. However, reporting did not necessarily mean a change would result; sometimes, queer asylum seekers were asked to endure.* (Wimark, 2021)	14 %
Some utilized harmful strategies, including substance abuse (Golembe et al., 2020; Oren & Gorshkov, 2021) or self-harm (Golembe et al., 2020) to cope with stressful circumstances	*Some participants tried to cope with their internalizing symptoms through substance abuse and self-harm. "Whatever drug I find I take, so I'd forget I'm living this life."* (Golembe et al., 2020)	7 %
Some purposefully decided to withdraw from their radicalized communities to have more space to live out their sexuality (Lee & Brotman, 2011)	*Some of the participants spoke about the complex relationship they had with their particular racialized community. One gay refugee described a purposeful decision to withdraw from his community in order to have more space to live out his sexuality.* (Lee & Brotman, 2011)	3 %

**Category: Psychological distress and manifestations** (Akin, 2017; Alessi, 2016; Alessi et al., 2018, 2020; Cerezo et al., 2014; Golembe et al., 2020; Held, 2022; Kahn, 2015a, 2015b; Kahn et al., 2018; Kahn & Alessi, 2018; Karimi, 2020a, 2021; Kostenius et al., 2021; Lee & Brotman, 2011; Llewellyn, 2021; Logie et al., 2016; Mulé, 2021; Murray, 2014a, 2014b; Novitskaya, 2021; Oren & Gorshkov, 2021; Rosati et al., 2021; Wimark, 2019, 2021)		**86 %**
Sub-category: Worries and fears (Alessi et al., 2018, 2020; Golembe et al., 2020; Kahn, 2015a, 2015b; Kostenius et al., 2021; Lee & Brotman, 2011; Llewellyn, 2021; Mulé, 2021; Murray, 2014b; Oren & Gorshkov, 2021; Wimark, 2019, 2021)		45 %
Migrants experienced several worries and fears what their future would entail, including:		
Potential social consequences and rejection when deciding to live openly or if exposed as LGBTQ+ (Golembe et al., 2020; Kahn, 2015a; Llewellyn, 2021; Mulé, 2021; Murray, 2014b; Oren & Gorshkov, 2021; Wimark, 2019)	*Several participants revealed that they avoid coming out to their friends as they fear the consequences. This behavior could be interpreted as rejection expectation. For instance, use of the word "gay" as a self-ascription in seemingly safe situations was challenging for some participants*. (Golembe et al., 2020)	24 %
The risk of being deported and relocated (Alessi et al., 2020; Kostenius et al., 2021; Lee & Brotman, 2011; Llewellyn, 2021)	*Participants lived in fear of being sent back to their home country or meeting the 'wrong people' at an interview at Migrationsverket [the Migration Authority]: "I'm still under migration, I'm still under investigation" (Miro), and "migration know (my) story … you know, in the interview [I] say what happened. Still, they want to know details like when they raped [me], what they were saying when they were raping you. It's hard, I mean … they rape me … I told them they hit me, everything they did to me, but like more and more details, it's not so easy. And when they ask, it's so hard you know, and they say, 'We need a strong story', like more, I say, 'What more?', they broke my arm, they had me one month, they raped me … what more can I say?"* (Kostenius et al., 2021)	14 %
Not having adequate protection and rights, not finding affirming services (Alessi et al., 2018, 2020)	*Participants also expressed that they worried about finding translators, immigration attorneys, and refugee-service providers who were LGBTQ-affirmative. Joshua described a situation that occurred by chance, which eventually enabled him to access the LGBTQ-affirmative services he needed*. (Alessi et al., 2018)	7 %
The risk of being exposed to discrimination and violence (Wimark, 2021)	*Moreover, the interviews showed signs of how the accommodation became a subtle place of fear. "I was in a straight camp and I was afraid of staying there, because I heard of the treatment of gays in the camp. Many times I asked at the reception in the camp to put me alone in a room, but they told me it is crowded and no places."* (Wimark, 2021)	3 %
Losing their ethnic identity and understanding of self (Oren & Gorshkov, 2021)	*The migration brought the issues of grappling with identities to the forefront for some, while others acknowledged that they still carried their "old" "Russianness" as solace and feared losing their ethnic identity and understanding of self*. (Oren & Gorshkov, 2021)	3 %
Sub-category: Loneliness and social exclusion (Akin, 2017; Alessi, 2016; Alessi et al., 2018; Golembe et al., 2020; Held, 2022; Karimi, 2020a; Lee & Brotman, 2011; Llewellyn, 2021; Logie et al., 2016; Mulé, 2021; Oren & Gorshkov, 2021; Rosati et al., 2021; Wimark, 2019)		45 %
A significant challenge was loneliness and social exclusion, particularly prominent when living in refugee centers, interacting in queer communities, waiting for asylum decision, and living undocumented. Insufficient language skills, limited social networks, not feeling ready to tell their stories, and being unemployed also contributed to social exclusion (Akin, 2017; Alessi, 2016; Alessi et al., 2018; Golembe et al., 2020; Held, 2022; Karimi, 2020a; Lee & Brotman, 2011; Llewellyn, 2021; Logie et al., 2016; Mulé, 2021; Oren & Gorshkov, 2021; Rosati et al., 2021; Wimark, 2019)	*Regardless of whether participants arrived in Austria or the Netherlands, many of the camps were far from cities with visible LGBTQ communities. This made it difficult for participants to meet other LGBTQ individuals, which contributed to their loneliness and isolation*. (Alessi et al., 2018)	45 %
Some deliberately isolated themselves due to mental exhaustion (Golembe et al., 2020)	*"In the city I am in, when I am in it, I don't interact with anyone, I don't go out with anyone. My psychological state is very tired, very tired."* (Golembe et al., 2020)	3 %
Sub-category: Symptoms of post-traumatic stress (Alessi, 2016; Kahn & Alessi, 2018; Karimi, 2021; Kostenius et al., 2021; Lee & Brotman, 2011; Llewellyn, 2021; Mulé, 2021)		24 %
Symptoms of post-traumatic stress were described, often triggered during asylum hearings when prompted to retell memories and previous exposure to violence in original country (Alessi, 2016; Kahn & Alessi, 2018; Karimi, 2021; Kostenius et al., 2021; Lee &	*The majority of participants reported that they experienced symptoms of PTSD upon their arrival to the host country and that these symptoms were connected to victimization experiences in their countries of origin*. (Alessi, 2016)	24 %

Brotman, 2011; Llewellyn, 2021; Mulé, 2021)		
Sub-category: Feelings of shame (Cerezo et al., 2014; Kahn, 2015a; Kahn et al., 2018; Lee & Brotman, 2011; Wimark, 2021)		17 %
Feelings of shame manifested related to previous trauma, sexual orientation and desires, relationships, their bodies, needing mental health services, and needing to wait for work permits (Cerezo et al., 2014; Kahn, 2015a; Kahn et al., 2018; Lee & Brotman, 2011; Wimark, 2021)	*Forced migrants observed that the stigma related to mental health care could be internalized as shame, making it diffcult for LGBT forced migrants to ask for help. In this regard, Quasim, a forced migrant who identi ed as gay, shared, "I don't know; I feel like [seeking mental health treatment] is also a negative thing to do, like self-blame."* (Kahn et al., 2018)	17 %
Sub-category: Other reactions and manifestations (Akin, 2017; Alessi, 2016; Alessi et al., 2018; Cerezo et al., 2014; Golembe et al., 2020; Held, 2022; Kahn, 2015a; Karimi, 2020a; Kostenius et al., 2021; Lee & Brotman, 2011; Llewellyn, 2021; Logie et al., 2016; Mulé, 2021; Murray, 2014a; Novitskaya, 2021; Oren & Gorshkov, 2021; Rosati et al., 2021)		59 %
A range of other psychological reactions and manifestations were described, including:		
Feeling depressed (Alessi, 2016; Golembe et al., 2020; Karimi, 2020a; Llewellyn, 2021; Logie et al., 2016; Oren & Gorshkov, 2021)	*She continued to suffer a great deal of depression, and she still felt isolated, even after she moved to a new living situation with a family who knows that she is gay.* (Llewellyn, 2021)	21 %
Anxiety (Alessi, 2016; Golembe et al., 2020; Llewellyn, 2021; Mulé, 2021; Murray, 2014a)	*Half of the interviewees were waiting in the pre-2018 queue, while the other half were subject to the new compressed post-2018 timeline. Both systems produced mental distress, as all interviewees reported problems sleeping and seeking out psychological services to deal with both their previous trauma and ongoing anxiety around the uncertainty of their future.* (Llewellyn, 2021)	17 %
Feeling overwhelmed (Alessi, 2016; Alessi et al., 2018; Murray, 2014a; Oren & Gorshkov, 2021)	*Experiencing numerous challenges in the host country contributed to participants feeling overwhelmed, depressed, and even desperate at times.* (Alessi, 2016)	14 %
Feeling questioned (Held, 2022; Kostenius et al., 2021; Murray, 2014a)	*Prince Emrah (Germany) said that their gender identity is often questioned. "In the clubs it's the same, they come, and they ask me, are you a girl or a boy?".* (Held, 2022)	10 %
Frustration (Akin, 2017; Lee & Brotman, 2011; Oren & Gorshkov, 2021)	*Eight participants expressed frustration about a long and unpredictable asylum process: [Sasha] "We have no guarantees. It's hard to imagine how much stress this is. Those who have not experienced this will not know; those who have not come here do not yet know that this is terrible stress."* (Oren & Gorshkov, 2021)	10 %
Anger (Cerezo et al., 2014; Rosati et al., 2021)	*The passages below elucidate the serious challenges women faced as a result of arriv-ing to the United States as transgender women. Sara, a 36-year-old woman of Mexican origin, reported: "Say they ask me for my name… a legal document that says, "Sara." Well, I don't have it. But I consider myself to be Sara and legally, well… but in me, I am Sara, but to get work you have to give a legal name, with legal identification. And well I have my Mexican papers, which has my legal name, and… it makes me so angry!"* (Cerezo et al., 2014)	7 %
Exhaustion (Golembe et al., 2020; Novitskaya, 2021)	*On some days I am too tired to go to school. And I don't because I am exhausted.* (Golembe et al., 2020)	7 %
Loss of control (Mulé, 2021; Oren & Gorshkov, 2021)	*A sense of freedom quickly transformed into a certain loss of control in the absence of guidance and support for new immigrants who had to start anew.* (Oren & Gorshkov, 2021)	7 %
Feeling skeptical and unsure (Kostenius et al., 2021; Mulé, 2021)	*The complexity of migration pro- cess offered reason to be sceptical and unsure of the future.* (Kostenius et al., 2021)	7 %
Feeling trapped (Akin, 2017; Llewellyn, 2021)	*Mahmut is frustrated, and feels trapped as an asylum seeker impatiently waiting for his appeal.* (Akin, 2017)	7 %
Hopelessness (Golembe et al., 2020; Karimi, 2020a)	*My interviews revealed that the combination of several factors including separation from family, ongoing social marginalization, dissatisfaction with employment status and earnings, and inability to plan for the future, has resulted in prevalent feelings of depression, isolation, and hopelessness among gay Iranian refugees.* (Karimi, 2020a)	7 %
Sleep disturbances (Llewellyn, 2021; Mulé, 2021)	*Half of the interviewees were waiting in the pre-2018 queue, while the other half were*	7 %

	subject to the new compressed post-2018 timeline. Both systems produced mental distress, as all interviewees reported problems sleeping and seeking out psychological services to deal with both their previous trauma and ongoing anxiety around the uncertainty of their future. (Llewellyn, 2021)	
Caught in no-mans land or limbo (Kostenius et al., 2021)	*Their experiences included being in limbo throughout the migration process.* (Kostenius et al., 2021)	3 %
Feeling desperate (Alessi, 2016)	*Experiencing numerous challenges in the host country contributed to participants feeling overwhelmed, depressed, and even desperate at times.* (Alessi, 2016)	3 %
Feeling out-of-place (Kostenius et al., 2021)	*Some participants seeking asylum described how they had a constant feeling of being questioned and out of place in their new lives.* (Kostenius et al., 2021)	3 %
Grief (Kahn, 2015a)	*Grief over strained or estranged relationships with kin in home and/or host countries persisted over time.* (Kahn, 2015a)	3 %
Guilt (Cerezo et al., 2014)	*Cynthia, a 40-year-old woman of Mexican origin, described the impact of violence from family and community members on her mental health and sense of self. She also described how her mental health provider was providing support to increase her self-esteem. "I feel ashamed, I feel guilty for what happened in my life. Rape, maltreatment, and all that... today I met with my therapist and she told me, "try to let everything out because it is not good for you." I tried to let everything out but sometimes, I can't. I want to cry, let it all out, and everything like that."* (Cerezo et al., 2014)	3 %
Hurt (Rosati et al., 2021)	*Ayoub reported two episodes of discrimination: one at their local Provincial Police Bureau and the other at a Provincial Centre for Adult Education: "So I've experienced it a lot in the institutional office–let's say official–in the police station, in the hospital, in the school, in the municipality, in the post office, everywhere–in the revenue agency, of course. [...] Everything [happened]. When you present yourself with your passport with a feminine personality and appear masculine, they ask you: 'So you want to be a woman? Are you male and want to become a woman? Ah ok you used to be a woman, so people like you exist too. Why did you change your mind when you arrived in Italy?' [...] Then I felt anger, I felt hurt, I felt maybe the first time that I feel equal to an Italian trans citizen because there is all trans people even if Italian citizens–the same things and... basically I was angry, I said I want to talk to the school manager, 'Don't you dare to say something like that in front of... ', I started to shout. However, he didn't do anything to me, they didn't do anything to me. I didn't take my right anyone defended me not... not even those of the school manager, he was very vulgar that person and indeed he looked at me bad all the school year."* (Rosati et al., 2021)	3 %
Psychological distress [without further specification] (Cerezo et al., 2014)	*Henrietta, a 32-year old woman of Mexican origin, shared how a history of violence related to transgender identity has contributed to bouts of severe psychological distress.* (Cerezo et al., 2014)	3 %
Sadness (Alessi, 2016)	*Participants also reported anxiety and sadness that were related to the complexities of resettlement in the United States and Canada.* (Alessi, 2016)	3 %

**Category: Practical issues related to resettlement and living conditions** (Akin, 2017; Alessi, 2016; Alessi et al., 2018, 2020; Cerezo et al., 2014; Golembe et al., 2020; Held, 2022; Kahn, 2015a; Kahn & Alessi, 2018; Karimi, 2020a; Lee & Brotman, 2011; Llewellyn, 2021; Logie et al., 2016; Mulé, 2021; Murray, 2014a, 2014b; Novitskaya, 2021; Oren & Gorshkov, 2021; Wimark, 2019)		**66 %**
Sub-category: Legal issues (Akin, 2017; Alessi, 2016; Kahn, 2015a; Kahn & Alessi, 2018; Lee & Brotman, 2011; Llewellyn, 2021; Mulé, 2021; Murray, 2014a, 2014b; Novitskaya, 2021; Oren & Gorshkov, 2021)		38 %
Dealing with legal issues was necessity when applying for asylum, sometimes involving difficulties securing the needed representation (Alessi, 2016; Kahn, 2015; Kahn & Alessi, 2018; Lee & Brotman, 2011; Murray, 2014; Oren & Gorshkov, 2021)	*Both service providers and LGBT forced migrants acknowledged the difficulties in navigating the refugee claims process, including securing legal representation, completing the persecution narrative and disclosing extremely private information*. (Kahn & Alessi, 2018)	21 %
Being tasked to provide a convincing case by providing confirming documents and detailed intimate descriptions was a difficult challenge (Akin, 2017; Kahn & Alessi, 2018; Llewellyn, 2021; Murray, 2014; Novitskaya, 2021; Oren & Gorshkov, 2021)	*The singular importance of 'proving' SOGI infused the transcripts. In countries with little or no protection for sexual and gender minorities, public disclosure or documentation of identities could present serious endangerment for LGBT individuals. Therefore, when LGBT refugee claimants are asked to provide documentation from the country of origin, they may have serious hesitation. Zoya, a forced migrant who identifies as lesbian, stated: "I was like, 'Well, how do I do that? There's no documents. Where am I going to get a document? I can't ask my family. I can't ask my friends. None of them know.' You know? And I would be worse off if I had gone somewhere in my country to get a piece of paper saying it was true*." (Kahn & Alessi, 2018)	21 %
To strengthen their claims, migrants felt a need to deepen their connections with the LGBTQ+ community, go public about their stories, and change their behaviors according to the ideas and norms of LGBTQ+ that case workers expect (Akin, 2017; Murray, 2014)	*A frequent application of rainbow splash manifests itself when some informants seek to go public in a strategic manner through social media, newspapers and interaction with particular queer organisations in order to communicate their sexual orientation and sometimes produce evidence for their alleged sexual identity*. (Akin, 2017)	7 %
Some encountered disbelief and poor translation services during the asylum process, which hindered a successful hearing (Akin, 2017)	*Kazim is complaining about the bad translation the interpreter provided during his asylum interview. It is this linguistic handicap, according to Kazim, that prevented him from expressing himself correctly to the caseworker and therefore led to the denial of his asylum claim*. (Akin, 2017)	3 %
As a last resort, some contemplated on producing photos of sexual acts, illustrating the extent they were committed to stay (Akin, 2017)	*In the absence of proper linguistic translation, Kazim seeks to follow another means of translation that he believes will facilitate his communication with the decision makers. He is contemplating over whether to produce photos of himself with another man while having sex. The staging of the sexual position, which would display Kazim as being penetrated, implies that his claim for genuineness – similar to Tooraj's overwhelming emphasis on being effeminate and 'bottom' – relies on his passivity in same-sexual conduct*. (Akin, 2017)	3 %
Transgender migrants encountered structural barriers when not being able to change their names during the asylum process (Lee & Brotman, 2011)	*Throughout the refugee determination process, trans refugees encountered particular structural barriers related to the inability to legally change their name on documentation*. (Lee & Brotman, 2011)	3 %
Sub-category: Difficulties upholding a financial situation (Alessi, 2016; Alessi et al., 2020; Cerezo et al., 2014; Held, 2022; Karimi, 2020a; Lee & Brotman, 2011; Llewellyn, 2021; Mulé, 2021; Murray, 2014b; Oren & Gorshkov, 2021; Wimark, 2019)		38 %
Securing employment needed to meet sufficient financial demands was a demanding process, largely because of intersectional bias and discrimination among employers (Cerezo et al., 2014; Karimi, 2020; Lee & Brotman, 2011; Llewellyn, 2021; Mulé, 2021; Oren & Gorshkov, 2021)	*Participants reported a range of challenges with securing em- ployment in the United States. These challenges illuminated the compounded barriers transgender immigrants face—women con- tended with bias centered on their transgender, immigrant, and racial identity and also had to deal with the tangible barriers due to a lack of documentation to work in the United States and/or mismatched documentation (name and gender) from their home country*. (Cerezo et al., 2014)	21 %
Living in a financially unstable situation led migrants into feeling forced to taking on involuntary work, including involuntary sex work or engaging in unwanted relationships, to make ends meet (Alessi, 2016; Alessi et al., 2020; Cerezo et al., 2014; Oren &	*He decided to risk deportation and stay in the Netherlands, which required him to keep a low profile for 18 months (i.e., until the Dublin statute of limitations expires if an individual cannot be located). Because of this, he was ineligible for social welfare*	17 %

Gorshkov, 2021; Wimark, 2019)	*benefits and unauthorized to work. Hence, he reported that he engaged in sex work in order to survive*. (Alessi et al., 2020)	
Further contributing to unemployment, many needed to wait long periods before obtaining work permits allowing legal work (Alessi, 2016; Lee & Brotman, 2011; Llewellyn, 2021; Murray, 2014; Oren & Gorshkov, 2021)	*Some also described the anguish that came along with waiting to receive a decision about their case. For example, Marc first applied for asylum in the United States in 2008, but he was not granted it until 2013. Concerns that he did not meet the filing date (1 year in the United States, and 30–60 days in Canada) extended his case for many years. Because those seeking refugee/asylee status are not legally authorized to work, he struggled financially and worried constantly about his future*. (Alessi, 2016)	17 %
Transgender migrants had to deal with conflicting documents when their presented gender identity and preferred pronouns did not match their names in certain official documents, leaving them in problematic predicaments (Cerezo et al., 2014)	*The issue of documentation was especially poignant here as women's legal name, gender on record, and preferred pronouns were not reflected on their home country identification. This placed women in especially challenging predicaments because working with false identification would have complicated their ability to seek asylum based on transgender need where having a criminal record can be means for dismissal of application*. (Cerezo et al., 2014)	3 %
Sub-category: Housing (Alessi et al., 2020; Lee & Brotman, 2011; Llewellyn, 2021; Mulé, 2021; Murray, 2014a, 2014b; Oren & Gorshkov, 2021)		24 %
Some experienced complex challenges related to housing and a risk of becoming homeless, including threatening, controlling, discriminating, and questioning behaviors among landlords, needing to conceal their LGBTQ+ identity, and difficulties attaining affordable housing (Alessi et al., 2020; Lee & Brotman, 2011; Llewellyn, 2021; Mulé, 2021; Murray, 2014a, 2014b; Oren & Gorshkov, 2021)	*Yet, a few ended up in difficult living situations where they felt they had no choice but to endure anti-Muslim attitudes. Kenan, a gay male in Vienna, described the challenges he faced when he rented a room in a house with a family from the mainstream community. He explained that one member of this family was very controlling, frequently commenting on the time that he arrived home in the evenings and, most significantly for Kenan, forcing him to eat foods that were against his religion*. (Alessi et al., 2020)	24 %
Sub-category: Leaving behind a stable life situation (Akin, 2017; Alessi, 2016; Alessi et al., 2018; Golembe et al., 2020; Held, 2022; Oren & Gorshkov, 2021)		21 %
Resettling in a new country entailed giving up well-paying jobs and stable housing, which meant a loss of a stable socioeconomic situation and resulted in feeling hurt (Akin, 2017; Alessi, 2016; Alessi et al., 2018; Golembe et al., 2020; Held, 2022; Oren & Gorshkov, 2021)	*Raha, a lesbian woman, explained that she had a well-paying job and lived on her own before migration. Now she had little money, stayed in migration camps, and lived in apartments with strangers: "This is really something that [hurts] my feelings at the moment. Because I have no other way. I don't know the language . . . I have no idea what to do other than accept the situation."* (Alessi et al., 2018)	21 %
Sub-category: Accessing healthcare (Logie et al., 2016; Mulé, 2021; Oren & Gorshkov, 2021)		10 %
Challenges related to not having adequate access to basic healthcare services were experienced, including difficulties navigating the health care system and not understanding what services that were covered, which contributed to poor health status and financial instability (Logie et al., 2016; Mulé, 2021; Oren & Gorshkov, 2021)	*Limitations on access to health services can seriously impact on both physical and mental health: "Last time I went round and round looking for a doctor. I was so sick. I had to take a taxi and I went round and round looking for a doctor to take care of me. Sick. I couldn't even walk. I don't know."* (Mulé, 2021)	10 %
Sub-category: Language barriers (Lee & Brotman, 2011; Mulé, 2021; Oren & Gorshkov, 2021)		10 %
Migrants experienced difficulties when having to deal with language barriers (Lee & Brotman, 2011; Mulé, 2021; Oren & Gorshkov, 2021)	*In describing the main challenges they faced in their new country, four participants ranked them in the same order: first, difficulty finding free (pro bono) or low-cost legal assistance; second, finding time to learn English as most come with a very low level of English proficiency; third, material hardship during the first year when asylum seekers are generally not allowed to work; and four, trouble with finding survival jobs* (Oren & Gorshkov, 2021)	10 %
Sub-category: School (Mulé, 2021; Novitskaya, 2021)		7 %
Migrants described little possibilities of applying for school, resulting in frustration (Mulé, 2021; Novitskaya, 2021)	*He continued to work at a Russian-speaking medical company in Brooklyn and was still waiting for his asylum interview date. Remembering that Friedrich's dream was to go back to school to become a nurse, I asked if there had been any progress on that front. "No," he responded, with evident frustration. "I wanted to enroll in a [community college] program, but without refugee status, they won't give me a resident tuition rate. To them, I am international student, and I can't afford that rate. It's such discrimination! I pay taxes like any other resident."* (Novitskaya, 2021)	7 %

Sub-category: Acquiring food (Alessi, 2016)		3 %
Some described having difficulties acquiring sufficient food for the day (Alessi, 2016)	*Lucy had to contend with difficult living conditions, too. Im- mediately following her arrival in the United States, the pastor of one of her family members found her a temporary place to stay with a group of women she had never met before. She was worried about them finding out that she was HIV-positive and that she was taking medication. The situation was also complicated by the fact that Lucy did not have much food to eat: "The lady who was always there because the other one was always working. . . . She was not always very happy seeing me in the kitchen. Of course I was not bring in any dime [laughs]. I really didn't get to eat. But why am I saying this? I'm saying this because that really, for the first time people have always talked about [medication] and food. That made me realized how nutrition is important when you're taking your [medication], because taking the pill without food, on an empty stomach can really . . . bring a lot of terrible side effects."* (Alessi, 2016)	3 %
Sub-category: Social services (Oren & Gorshkov, 2021)		3 %
Some described not having access to adequate social services (Oren & Gorshkov, 2021)	*The participants described conflicting feelings between the initial euphoric stage during the first several months in a new country that some participants experienced regarding safety and freedom and the later discovery that social service support was disjointed, fragmented, and often inaccessible.* (Oren & Gorshkov, 2021)	3 %

References

Akin, D. (2017). Queer asylum seekers: Translating sexuality in Norway. *Journal of Ethnic and Migration Studies*, *43*(3), 458–474. https://doi.org/10.1080/1369183X.2016.1243050

Alessi, E. J. (2016). Resilience in sexual and gender minority forced migrants: A qualitative exploration. *Traumatology*, *22*(3), 203–213. psyh. https://doi.org/10.1037/trm0000077

Alessi, E. J., Greenfield, B., Kahn, S., & Woolner, L. (2021). (Ir)reconcilable identities: Stories of religion and faith for sexual and gender minority refugees who fled from the Middle East, North Africa, and Asia to the European Union. *Psychology of Religion and Spirituality*, *13*(2), 175–183. psyh. https://doi.org/10.1037/rel0000281

Alessi, E. J., Kahn, S., Greenfield, B., Woolner, L., & Manning, D. (2020). A qualitative exploration of the integration experiences of LGBTQ refugees who fled from the Middle East, North Africa, and Central and South Asia to Austria and the Netherlands. *Sexuality Research & Social Policy: A Journal of the NSRC*, *17*(1), 13–26. psyh. https://doi.org/10.1007/s13178-018-0364-7

Alessi, E. J., Kahn, S., Woolner, L., & Van Der Horn, R. (2018). Traumatic Stress Among Sexual and Gender Minority Refugees From the Middle East, North Africa, and Asia Who Fled to the European Union. *Journal of Traumatic Stress*, *31*(6), 805–815. https://doi.org/10.1002/jts.22346

Cerezo, A., Morales, A., Quintero, D., & Rothman, S. (2014). Trans migrations: Exploring life at the intersection of transgender identity and immigration. *Psychology of Sexual Orientation and Gender Diversity*, *1*(2), 170–180. psyh. https://doi.org/10.1037/sgd0000031

Dhoest, A. (2019). Learning to be gay: LGBTQ forced migrant identities and narratives in Belgium. *Journal of Ethnic and Migration Studies*, *45*(7), 1075–1089. Scopus. https://doi.org/10.1080/1369183X.2017.1420466

Dhoest, A. (2020). Digital (dis)connectivity in fraught contexts: The case of gay refugees in Belgium. *European Journal of Cultural Studies*, *23*(5), 784–800. Scopus. https://doi.org/10.1177/1367549419869348

Golembe, J., Leyendecker, B., Maalej, N., Gundlach, A., & Busch, J. (2020). Experiences of Minority Stress and Mental Health Burdens of Newly Arrived LGBTQ* Refugees in Germany. *Sexuality Research and Social Policy*. https://doi.org/10.1007/s13178-020-00508-z

Held, N. (2022). “As queer refugees, we are out of category, we do not belong to one, or the other”: LGBTIQ+ refugees’ experiences in “ambivalent” queer spaces. *Ethnic and Racial Studies*, 1–21. https://doi.org/10.1080/01419870.2022.2032246

Kahn, S. (2015a). Cast out: “Gender Role Outlaws” seeking asylum in the west and the quest for social connections. *Journal of Immigrant and Refugee Studies*, *13*(1), 58–79. Scopus. https://doi.org/10.1080/15562948.2014.894169

Kahn, S. (2015b). Experiences of Faith for Gender Role Non-Conforming Muslims in Resettlement: Preliminary Considerations for Social Work Practitioners. *British Journal of Social Work*, *45*(7), 2038–2055. cin20. https://doi.org/10.1093/bjsw/bcu060

Kahn, S., & Alessi, E. J. (2018). Coming out under the gun: Exploring the psychological dimensions of seeking refugee status for lgbt claimants in Canada. *Journal of Refugee Studies*, *31*(1), 22–41. Scopus. https://doi.org/10.1093/jrs/fex019

Kahn, S., Alessi, E. J., Kim, H., Woolner, L., & Olivieri, C. J. (2018). Facilitating mental health support for LGBT forced migrants: A qualitative inquiry. *Journal of Counseling & Development*, *96*(3), 316–326. psyh. https://doi.org/10.1002/jcad.12205

Karimi, A. (2020a). Limits of Social Capital for Refugee Integration: The Case of Gay Iranian Male Refugees’ Integration in Canada. *International Migration*, *58*(5), 87–102. Scopus. https://doi.org/10.1111/imig.12691

Karimi, A. (2020b). Refugees’ Transnational Practices: Gay Iranian Men Navigating Refugee Status and Cross-border Ties in Canada. *Social Currents*, *7*(1), 71–86. Scopus. https://doi.org/10.1177/2329496519875484

Karimi, A. (2021). Sexuality and integration: A case of gay Iranian refugees’ collective memories and integration practices in Canada. *Ethnic and Racial Studies*, *44*(15), 2857–2875. https://doi.org/10.1080/01419870.2018.1550207

Kostenius, C., Hertting, K., Pelters, P., & Lindgren, E.-C. (2021). From Hell to Heaven? Lived experiences of LGBTQ migrants in relation to health and their reflections on the future. *Culture, Health & Sexuality*, 1–13. https://doi.org/10.1080/13691058.2021.1983020

Lee, E. O. J., & Brotman, S. (2011). Identity, refugeeness, belonging: Experiences of sexual minority refugees in Canada. *Canadian Review of Sociology = Revue Canadienne de Sociologie*, *48*(3), 241–274. https://doi.org/10.1111/j.1755-618x.2011.01265.x

Llewellyn, C. (2021). Captive While Waiting to Be Free: Legal Violence and LGBTQ Asylum Applicant Experiences in the USA. *Sexuality Research and Social Policy*, *18*(1), 202–212. Scopus. https://doi.org/10.1007/s13178-020-00449-7

Logie, C. H., Lacombe-Duncan, A., Lee-Foon, N., Ryan, S., & Ramsay, H. (2016). "It's for us -newcomers, LGBTQ persons, and HIV-positive persons. You feel free to be": A qualitative study exploring social support group participation among African and Caribbean lesbian, gay, bisexual and transgender newcomers and refugees in Toronto, Canada. *BMC International Health and Human Rights*, *16*(1), 18. https://doi.org/10.1186/s12914-016-0092-0

Mulé, N. J. (2021). Mental health issues and needs of LGBTQ+ asylum seekers, refugee claimants and refugees in Toronto, Canada. *Psychology and Sexuality*. Scopus. https://doi.org/10.1080/19419899.2021.1913443

Murray, D. A. B. (2014a). Real queer: "Authentic" LGBT refugee claimants and homonationalism in the Canadian Refugee System. *Anthropologica*, *56*(1), 21–32. psyh.

Murray, D. A. B. (2014b). The challenge of home for sexual orientation and gendered identity refugees in Toronto. *Journal of Canadian Studies*, *48*(1), 132–152. Scopus. https://doi.org/10.1353/jcs.2014.0019

Novitskaya, A. (2021). Sexual Citizens in Exile: State-Sponsored Homophobia and Post-Soviet LGBTQI+ Migration. *Russian Review*, *80*(1), 56–76. Scopus. https://doi.org/10.1111/russ.12298

Oren, T., & Gorshkov, A. (2021). Lived Experiences of Recent Russian-Speaking LGBT+ Immigrants in the United States: An Interpretive Phenomenological Analysis. *Journal of LGBT Issues in Counseling*, *15*(3), 290–309. cin20. https://doi.org/10.1080/15538605.2021.1914278

Rosati, F., Coletta, V., Pistella, J., Scandurra, C., Laghi, F., & Baiocco, R. (2021). Experiences of Life and Intersectionality of Transgender Refugees Living in Italy: A Qualitative Approach. *International Journal of Environmental Research and Public Health*, *18*(23). https://doi.org/10.3390/ijerph182312385

Wimark, T. (2019). Homemaking and perpetual liminality among queer refugees. *Social and Cultural Geography*. Scopus. https://doi.org/10.1080/14649365.2019.1619818

Wimark, T. (2021). Housing policy with violent outcomes–the domestication of queer asylum seekers in a heteronormative society. *Journal of Ethnic and Migration Studies*, *47*(3), 703–722. Scopus. https://doi.org/10.1080/1369183X.2020.1756760
