# Supplementary material for: Post‐migration psychosocial experiences and challenges amongst LGBTQ+ forced migrants: A meta‐synthesis of qualitative reports
Source: J Adv Nurs. 2022 Nov 1;79(1):358–71. doi: 10.1111/jan.15480 (PMC10092230; doi:10.1111/jan.15480)
Supplement: Supplementary file 6 — File 6 [file JAN-79-358-s005.pdf]

Additional File 6. Summary of the synthesis in the theme Finding self-acceptance and tools utilized for resilience and strength in a social context with new possibilities, with frequency effect sizes (FES).

CATEGORY, SUB-CATEGORY AND CONTENT	ILLUSTRATIVE QUOTE	FES
**Category: Resilience and strengthening resources** (Alessi, 2016; Alessi et al., 2018, 2020, 2021; Cerezo et al., 2014; Dhoest, 2019, 2020; Golembe et al., 2020; Held, 2022; Kahn, 2015a, 2015b; Kahn et al., 2018; Karimi, 2020b, 2020a, 2021; Kostenius et al., 2021; Lee & Brotman, 2011; Logie et al., 2016; Mulé, 2021; Murray, 2014a, 2014b; Novitskaya, 2021; Oren & Gorshkov, 2021; Rosati et al., 2021; Wimark, 2019, 2021)		**90 %**
Sub-category: Appreciating the situation in the host country (Alessi, 2016; Alessi et al., 2018, 2020; Dhoest, 2019, 2020; Golembe et al., 2020; Held, 2022; Karimi, 2021; Kostenius et al., 2021; Logie et al., 2016; Murray, 2014a, 2014b; Novitskaya, 2021; Oren & Gorshkov, 2021)		48 %
Migrants expressed great appreciation of having the opportunity to live as their authentic selves, which resulted in a sense of relief, excitement, optimism, freedom, and enhanced self-acceptance (Alessi, 2016; Alessi et al., 2018, 2020; Dhoest, 2019, 2020; Golembe et al., 2020; Held, 2022; Karimi, 2021; Kostenius et al., 2021; Logie et al., 2016; Murray, 2014a, 2014b; Novitskaya, 2021; Oren & Gorshkov, 2021)	*Migrants experienced relief when they found they were able to be open about their sexual orientation and be true to themselves, no longer having to be silent or hide for fear of harassment and oppression. As Grace described it, Society is okay, they are accepting, they're not judgemental ... you're not going to hide like 'Oh, I have to hide about this' 'Oh I have to'—no, here you live your life, you live freely. So, we're not living in fear like we used to ... yeah.* (Kostenius et al., 2021)	48 %
When contrasting the difference in pre-migration and post-migration hardships, migrants described an improved overall situation (Dhoest, 2019, 2020; Murray, 2014b; Novitskaya, 2021; Oren & Gorshkov, 2021)	*What emerges, in this and all other interviews with refugees, is a clear opposition between the situation in Belgium and that in their home country. All describe a difficult 'before' (in their home country) and a much improved 'after' (in Belgium).* (Dhoest, 2020)	17 %
Through the increased legal protection and by living in a different social context allowing LGBTQ+ identities, they could lead a life without the same strong fear of harassment and oppression as experienced in their country of origin (Dhoest, 2019, 2020; Held, 2022; Murray, 2014a; Novitskaya, 2021)	*They appreciate the freedom and state protection they enjoy in Belgium, as 'out' gay men, and they have very limited experiences with homophobia and overt racism* (Dhoest, 2020)	17 %
Migrants expressed new opportunities for employment in host country (Novitskaya, 2021)	*As an asylum-seeker with a pending application, he can secure a work permit and find employment to pay his rent and bills*. (Novitskaya, 2021)	3 %
Sub-category: Peer support and LGBTQ+ organizations (Alessi, 2016; Alessi et al., 2020; Cerezo et al., 2014; Held, 2022; Kahn, 2015b; Karimi, 2020b, 2020a; Kostenius et al., 2021; Lee & Brotman, 2011; Logie et al., 2016; Murray, 2014a, 2014b; Oren & Gorshkov, 2021; Wimark, 2019)		48%
By interacting with peers, migrants developed meaningful and strong relationships with likeminded and understanding companions in a safe and accepting setting (Alessi, 2016; Alessi et al., 2020; Cerezo et al., 2014; Held, 2022; Karimi, 2020a; Kostenius et al., 2021; Lee & Brotman, 2011; Logie et al., 2016; Murray, 2014a, 2014b; Oren & Gorshkov, 2021; Wimark, 2019).	*Others discussed feeling a sense of belonging and kinship in Canada after attending the support groups: "It's sort of finding a family. When I came here, it was winter time. Everybody was in their own house. It was like, oh, there is no life. But the day I met [group A], it was like chatting, laughing. It made me feel welcome in Canada."* (Logie et al., 2016)	41 %
Through peer support activities, migrants established trust and found a social setting in which they felt they belonged, breaking social isolation, exclusion, and structural marginalization (Alessi et al., 2020; Held, 2022; Karimi, 2020a; Kostenius et al., 2021; Logie et al., 2016; Murray, 2014a, 2014b; Oren & Gorshkov, 2021).	*Most of the interviewees spoke about the vital importance of LGBT refugee support groups—for many, this was the first time they had been in a safe space with ''people like me'' (Olu), where they could talk with each other, find out information about what was expected at hearings and how other lawyers were treating their clients, what jobs other refugees were finding and other issues pertaining to the refugee determination process and settlement*. (Murray, 2014a)	28 %
Peer support involved informational support, as peers shared valuable information and knowledge needed to survive and thrive in the host country, such as how to apply for asylum and work permits, learning languages, navigating the migration process, and learning about sexual health and relationships (Alessi, 2016; Kostenius et al., 2021; Logie et al., 2016; Murray, 2014a, 2014b; Oren & Gorshkov, 2021; Wimark, 2019).	*Moreover, interactions with community providers could result in new information crucial to participants' survival in the host coun- try. For example, Eduard came to the United States on a student visa and worried about returning to his country of origin after it expired. He learned about asylum after attending a support group for LGBT immigrants*. (Alessi, 2016)	24 %
Instrumental support occurred in peer interactions, through the tangible assistance with financial help, networking, help attaining housing and legal assistance, support for substance abuse, and help securing employment (Alessi et al., 2020; Cerezo et al., 2014;	*Participants discussed how the support groups offered volunteer opportunities and employment workshops that helped them attain employment in Canada. One of the participants volunteered at the ASO described: "Through my volunteering here, I was*	24 %

Held, 2022; Logie et al., 2016; Murray, 2014a, 2014b; Oren & Gorshkov, 2021).	*able to get job references for the job that I have now. And even through their employment workshop, that also helped me pick up the job I have right now.”* (Logie et al., 2016)	
For many, peer support improved mental health, including enhancing confidence, self-worth, and identity acceptance, reducing feelings of shame, depression, and loneliness, and improving coping with stress and challenges (Alessi, 2016; Held, 2022; Karimi, 2020a; Kostenius et al., 2021; Lee & Brotman, 2011; Logie et al., 2016)	*Many participants described how their mental health improved after they began attending the support groups. One narrative reflected increased hope: “I love [the ASO]. The day I came, it was everything that was dead in me, it revived again. I started singing.”* (Logie et al., 2016)	21 %
Through peer support, migrants exchanged emotional support, contributing to feeling normalized, valued, less stressed and depressed, less lonely and isolated, and relief (Alessi, 2016; Cerezo et al., 2014; Kostenius et al., 2021; Logie et al., 2016; Murray, 2014b).	*A participant narrative articulated how emotional support helped reduce the stress of refugee claimants: “Those that have gone through [a refugee hearing] are usually a great encouragement to those who have not gone through it yet. And I can say, those who have not gone through are usually extremely stressed.”* (Logie et al., 2016)	17 %
Peer support led to migrants feeling strengthened and better equipped to push back structural barriers contributing to intersectional marginalization (Cerezo et al., 2014; Held, 2022; Kostenius et al., 2021; Lee & Brotman, 2011).	*Sexual minority refugees with stronger linkages with queer racialized communities, mainstream queer communities, and their particular racialized community were able to better push back against structural barriers and intersectional marginalizing experiences.* (Lee & Brotman, 2011)	14 %
When seeing peers thrive and meeting role models, migrants began to feel less fearful and more hopeful about their own future (Alessi, 2016; Logie et al., 2016; Oren & Gorshkov, 2021).	*One person talked about being turned to activism by RUSA: [Misha]” Well, it seems to me, for example, for me the role model in RUSA was [name], because he is so fearless, he is not afraid of anything. and then [name], I don’t know whether you know [name] or not, [name], [name], for me they … Actually, they helped me overcome my fears very much. To some extent, they are role models for me. And maybe my…, I and my actions, I really hope that I will be someone’s role model, and people will come, do come out and work, no longer afraid of any consequences.”* (Oren & Gorshkov, 2021)	10 %
Some felt unaware of peer support organizations or declined a need for such activities, and a few also discussed negative effects on their mental health when spending time with peers and LGBTQ+ organizations (Alessi et al., 2020; Kahn, 2015b).	*In contrast, while Mohammed, a gay male, felt grateful for the support he received from LGBTQ refugee organizations in Amsterdam, he expressed that he had little in common with LGBTQ individuals from the refugee and host communities. In fact, spending time with other LGBTQ individuals often left him feeling lonely and alienated: ”And when you go to such meetings in such organiza- tions, afterward, I could cry out of bitterness and lone- liness. When I go there, I feel even more lonely. Because I come from a homophobic country, I didn’t have any chance with my countrymen. And with these people the only thing I have in common with them is my sexual orientation, which is based on practically nothing.”* (Alessi et al., 2020)	7 %
Giving back and helping struggling peers by engaging in activism and volunteering strengthened resilience and involved:		
Not only did receiving peer support involve personal benefits, providing it to other peers was also rewarding and for many a driving force for resilience (Alessi, 2016; Cerezo et al., 2014; Held, 2022; Karimi, 2020b; Kostenius et al., 2021; Lee & Brotman, 2011; Logie et al., 2016; Oren & Gorshkov, 2021)	*For Helen, helping others enabled her to find strength in the midst of adversity. She recalled an incident in which she was fired from a job in Canada after coworkers discovered she was trans female. However, she maintained her resolve in knowing that others needed her.* (Alessi, 2016)	28 %
Migrants engaging peer support expressed that providing it to others and taking part in activism involved an opportunity to transcend their own difficult situations and was a welcomed distraction from their own hardships (Alessi, 2016).	*In their attempts to give back, participants were able to transcend their own difficulties, even if it was just for a short period of time. For example, John started his advocacy work in his country of origin and continues to be active in Canada. His advocacy work helped strengthen his resilience.* (Alessi, 2016)	3 %
Engaging in volunteer work and activism was seen as a meaningful endeavor, which resulted in feeling productive and like they were contributing to a greater social change and awareness (Alessi, 2016; Cerezo et al., 2014; Held, 2022; Karimi, 2020b; Kostenius et al., 2021; Lee & Brotman, 2011; Logie et al., 2016; Oren & Gorshkov, 2021).	*I was invited by my participants to be a forum member of an online Iranian LGBT group whose members were liv- ing in Canada, the United States, and Turkey. I observed that several of my participants, who were also members of this forum, were involved in discussing and formulating strategies as well as guiding lobbying meetings and community politics with Canadian and American activists. Most of these efforts were targeted at increas- ing awareness about gay Iranian asylum seekers’ situations in Turkey and the necessity of expediting their case processing. The participant who invited*	28 %

	me to join the online group said, "The primary goal is to increase awareness... The Canadian government has put a halt on resettling LGBT refugees from Turkey, but most Canadian activists do know about this so we have been trying to arrange meetings with our MPs in different cities and with LGBT activist groups to talk about this issue and find ways to restart the program." (Karimi, 2020b)	
Sub-category: Social support from non-peers(Alessi, 2016; Alessi et al., 2020; Cerezo et al., 2014; Kahn, 2015a; Karimi, 2020a, 2021; Lee & Brotman, 2011; Mulé, 2021; Murray, 2014b; Oren & Gorshkov, 2021; Rosati et al., 2021; Wimark, 2019, 2021)		45 %
Some were able to find ways to coexist and receive support from their coethnic communities, leading to a successful integration of their LGBTQ+ identity and their cultural identity (Karimi, 2020a, 2021; Lee & Brotman, 2011; Murray, 2014b; Wimark, 2019, 2021).	This gay refugee described how, over time, he was able to negotiate his sexualty with his cultural identity and move toward affirmation. "I went to a party within my community... with my partner... and people knew and accepted me regardless... I never had any problems... this helped in my affirmation process so I could say to myself ''you can have a life with your partner'' and I can still belong to my community... its my culture, my identity. So if I can marry the two, that's good." (Lee & Brotman, 2011)	21 %
Members within the mainstream host community, including non-peer LGBTQ+ people, supported migrants to meet basic needs such as housing, legal assistance, and emotional support (Alessi et al., 2020; Kahn, 2015a; Karimi, 2020a; Lee & Brotman, 2011; Wimark, 2019); leading to mixed feelings involving both positive experiences but also feeling dependent and being exposed to potential exploitation and abuse (Alessi et al., 2020)	*During interviews I found that bridging social capital with, often White, non- co-ethnics had led participants to find jobs and learn about educational and training opportunities without having to confront the stigmas surrounding sexuality. For instance, one participant who was working as a trainer said "I was dating a Canadian guy and his friend helped me with finding and getting this job... I learn about many work opportunities and workshops on fitness training through my team members".* (Karimi, 2020a)	17 %
Migrants formed new chosen families and significant others while living in the host country, associated with several personal benefits for psychological wellbeing and quality of life (Alessi, 2016; Cerezo et al., 2014; Mulé, 2021).	*Chris also relied on his husband for support. His parents disowned him after finding out that he was engaged to a man, making him realize the importance of his family of choice: "Having a family that I sort of, a rat pack family who I put together. People who care and who are understanding. Who doesn't judge. Who um, I mean, I have an awesome husband."* (Alessi, 2016)	10 %
Some were welcomed in churches, which in addition to social support also involved a reparative path to faith (Oren & Gorshkov, 2021; Rosati et al., 2021).	*Maria (34-year-old transwoman of Cuban origin and Evangelical Pente- costal religion) experienced her gender affirmation path as a reparation and returning to "God's way": "Actually I didn't have a bad experience because I was lucky that the church I was attending and the leader of the church were saying that God welcomes everyone as we are and the same God changes you and you have to reshape yourself in God's way. That's why we had this problem."* (Rosati et al., 2021)	7 %
Sub-category: Religion, faith, and spirituality (Alessi, 2016; Alessi et al., 2021; Cerezo et al., 2014; Kahn, 2015b; Lee & Brotman, 2011; Rosati et al., 2021)		21 %
For some, religion, faith, and spirituality bolstered their resilience, increased their optimism, and enhanced their self-acceptance (Alessi, 2016; Alessi et al., 2021; Cerezo et al., 2014; Lee & Brotman, 2011; Rosati et al., 2021).	*Participants relied on religion and spirituality to bolster their resilience. Although a few participants identifying as White de- scribed that spirituality strengthened their resilience, this was particularly the case for African and Caribbean participants.* (Alessi, 2016)	17 %
Migrants turned to faith to persevere and cope with discrimination and bias by seeking comfort in God or another source (Cerezo et al., 2014).	*Belief that a higher power was responsible for creating transgender people facilitated participants' self- acceptance of their gender identity and helped them persevere when faced with transgender-specific bias and discrimination.* (Cerezo et al., 2014)	3 %
In some cases, the need to live out their faith and attend churches preceded their need to be open about the LGBTQ+ identity, which made them conceal their LGBTQ+ identity during religious activities (Alessi, 2016).	*For some, the desire for connection to God superseded the need to be open with their sexual orientation. For instance, Lucy re- ported that she attended a church that was close to her home and that she did not mind hiding her sexual orientation while there.* (Alessi, 2016)	3 %
Sub-category: Professional support (Alessi, 2016; Kahn, 2015a, 2015b; Kahn et al., 2018; Rosati et al., 2021)		17 %
Professional support from mental health services offered important tools for psychological adaptation and relief (Alessi, 2016; Kahn, 2015a, 2015b)	*After migrating to the United States and Canada, participants utilized community services to strengthen their resilience, with mental health services being the most common. Therapy helped participants to manage the psychological effects of severe*	10 %

	trauma. (Alessi, 2016)	
Coming in contact with adequate services was sometimes challenging, when migrants were unaware of available services or decided not to seek out professional support because of fear of shame or stigma (Alessi, 2016; Kahn, 2015a; Kahn et al., 2018)	*Forced migrants observed that the stigma related to mental health care could be internalized as shame, making it diffcult for LGBT forced migrants to ask for help*. (Kahn et al., 2018)	10 %
Besides health professionals, migrants also received professional support and established bonds with lawyers and social service workers (Alessi, 2016; Kahn, 2015a)	*Establishing supportive relationships with legal and/or psychosocial care providers in perimigration was typically experienced by study participants as a turning point, providing glimmers of hope and validation. For the ma- jority of study participants, forming these relationships was tantamount to recreating ruptured familial bonds*. (Kahn, 2015a)	7 %
Migrants emphasized the importance of being able to connect with and trust their counselor, which for some included meeting counselors with similar orientation or identity as themselves (Kahn et al., 2018)	*Zoya, a forced migrant participant who identi ed as lesbian, warned that gateway providers should not assume that merely locating a mental health professional from the LGBT community is sufficient. She encouraged providers to ask clients about the type of counselor with whom they feel comfortable working. Although she appreciated the opportunity to speak with a gay-identified counselor, she felt, nonetheless, that the gay male counselor could not fully understand her experiences: "I would prefer somebody who is a lesbian… as a counselor… I feel that a lesbian would be better able to relate to my issues and address my concerns*." (Kahn et al., 2018)	3 %
Trans migrants expressed a fundamental role of access to gender affirmation services (Rosati et al., 2021)	*In their individuation process, participants conferred a fundamental role to medical-surgical treatments for gender affirmation (e.g., access to hormones and surgery), which represented methods for bringing one's gender expression closer to one's gender identity*. (Rosati et al., 2021)	3 %
Sub-category: Being optimistic (Alessi, 2016; Alessi et al., 2018; Lee & Brotman, 2011)		10 %
Migrants emphasized the importance of remaining hopeful during long asylum processes, thinking optimistically about their future and not dwelling on their past, even when their situation meant a loss of dreams and aspirations or despite feeling isolated and dehumanized (Alessi, 2016; Alessi et al., 2018; Lee & Brotman, 2011)	*Marc discussed how he managed to stay resilient during this period in his life: "Try to think positive and try to think about the future, not about the past. Because this day will come, this day will come. I mean, all your sufferings will end. That's how I was trying to not to lose my mind [laughs]. I was hoping all the time…"* (Alessi, 2016)	
Sub-category: Staying committed (Alessi, 2016; Cerezo et al., 2014; Karimi, 2020a)		10 %
Migrants were committed on to taking jobs in host country and invested much time in securing employment, even when unfamiliar with the tasks it entailed or when having the option of staying at home (Alessi, 2016; Karimi, 2020a)	*Albert remained committed to doing whatever it took to survive in the host country until he could find more suitable employment: "I'm here for a year, I live by myself, I do not have a—I didn't get any roommates so far. I'm working at a [restaurant]. I have a very difficult job. I could stay at home and apply for disability or social assistance more and stay at home*." (Alessi, 2016)	7 %
The resourcefulness of migrants contributed to them being able to deal with bias and discrimination (Cerezo et al., 2014)	*It is important to emphasize how women's resourcefulness was the most prominent factor that contributed to their mental health and aided them when dealing with the varied and oftentimes overwhelming bias and discrimination they faced as transgender immigrant Latinas*. (Cerezo et al., 2014)	3 %

**Category: Identity formation** (Akin, 2017; Alessi et al., 2021; Cerezo et al., 2014; Dhoest, 2020; Golembe et al., 2020; Held, 2022; Kahn, 2015a, 2015b; Karimi, 2020b, 2020a, 2021; Lee & Brotman, 2011; Llewellyn, 2021; Logie et al., 2016; Murray, 2014a; Oren & Gorshkov, 2021; Rosati et al., 2021; Wimark, 2019)		**62 %**
Sub-category: Identity self-acceptance (Akin, 2017; Alessi et al., 2021; Dhoest, 2020; Golembe et al., 2020; Kahn, 2015b; Karimi, 2021; Lee & Brotman, 2011; Llewellyn, 2021; Logie et al., 2016; Oren & Gorshkov, 2021; Rosati et al., 2021; Wimark, 2019)		41 %
Migrants expressed enhanced self-acceptance by acknowledging all parts of their identity without labels and not feeling scared about it, while also allowing their identity to change over time (Golembe et al., 2020; Karimi, 2021; Lee & Brotman, 2011; Llewellyn, 2021; Logie et al., 2016; Rosati et al., 2021; Wimark, 2019).	*Finally, the third theme highlighted the importance that participants placed on rec- ognizing and defining themselves in gender categories that differed from those of the West. One participant used the Arabic term aber to refer to transgender people who were masculine or non-binary. Other participants rejected the transgender label in favor of an identification limited to perceived gender, which (in these cases) concerned the category of "woman"*. (Rosati et al., 2021)	24 %
Another part of their self-acceptance was reconciling their view of religion and faith; some decided to shun away from the religious doctrines that involved the norms they were forced to flee from, while others decided to redefine these structures in their own terms or continue to practice the same religion as before (Alessi et al., 2021; Kahn, 2015b; Oren & Gorshkov, 2021; Rosati et al., 2021).	*Remarkably, even participants who lacked affirmative religious experiences were able to use religion as a coping strategy, by developing theories about their gender experiences that diverged from traditional theology and religious frameworks, but allowed them to live out their spirituality in a positive way*. (Rosati et al., 2021)	14 %
Feeling free to express themselves in society, some migrants decided to live '*loud and proud*' of their LGBTQ+ identity, embracing a lifestyle not previously possible in their country of origin (Akin, 2017; Dhoest, 2020).	*Overall, however, they are 'proud' to be gay. For instance, Samuel says: "I never regretted being gay, I am still proud to be gay. Belgium gives me the opportunity for me to live the way I want to live my life. So that makes me feel proud, I live my life the way I want to"*. (Dhoest, 2020)	7 %
Sub-category: Identity formation through chosen or involuntary distancing of social contacts (Alessi et al., 2021; Dhoest, 2020; Kahn, 2015a, 2015b; Karimi, 2020a, 2021; Lee & Brotman, 2011; Llewellyn, 2021; Oren & Gorshkov, 2021; Wimark, 2019)		34 %
A strategy to avoid negative social consequences and achieve a higher level of personal freedom among some migrants was to restrict their communication with - or decide to altogether shun away from - heteronormative compatriots, family members, and relatives (Dhoest, 2020; Kahn, 2015a; Karimi, 2020a, 2021; Lee & Brotman, 2011; Oren & Gorshkov, 2021; Wimark, 2019)	*Although currently there are a few queer Muslim groups in major Canadian cities who embrace sexual minorities from Middle-Eastern backgrounds, none of the participants had experience of or desire for membership in such communities*. (Karimi, 2021)	24 %
Some rejected or relinquished their ties to traditional religious doctrines, experiencing personal distress and anguish caused by these religious structures (Alessi et al., 2021; Kahn, 2015b; Karimi, 2021)	*The lasting impacts of negative religious messaging in partici- pants' countries of origin led most (64.7%) to eventually reject Islam after their arrival in Austria or the Netherlands. For example, Adam, a gay male, expressed relief that he was now able to distance himself from his faith in the host country: "I never talk religion with anyone. And almost [all] of my friends, even the Dutch people, they are not [religious]. So, we never have these conversations."* (Alessi et al., 2021)	10 %
Some were reluctant to participate in certain LGBTQ+ related activities in host country (Karimi, 2021; Llewellyn, 2021; Oren & Gorshkov, 2021)	*Many LGBTQ applicants perceived prior to coming to the USA that the culture is welcoming and accepting of sexual minorities. However, most expressed reluctance at participating in LGBTQ events. While some attended large-scale events, like pride parades, few joined any organizations upon arrival in the USA*. (Llewellyn, 2021)	10 %
Some deliberately distanced themselves from certain news/information about their country of origin because they wanted to see governmental changes and are not interested in the oppressive situation for LGBTQ+ persons (Dhoest, 2020)	*The Internet is also the key source of information on their country of origin, but quite a few participants deliberately distance themselves from it. For instance, when asked if he uses the Internet to know what's happening in Sierra Leone, Samuel says, "Very little, I pay very little attention, because I am an activist now, and we want to see that the government takes responsibility and gives people their right."* (Dhoest, 2020)	3 %
Some distanced themselves from heterosexual persons, based on their sexual orientation and history of rejection (Karimi, 2020a)	*For my participants, as a minority within a diverse ethnic group, weak ties or social bridges were represented in ties with heterosexual co-ethnics and non-co-ethnics. Despite the presence of well- established Iranian communities in Canada, my participants were unwilling to reach out to community resources and social opportunities, since their non-confirming sexuality and histories of rejection inhibited the development of ethnicities*. (Karimi, 2020a)	3 %

Sub-category: Social influence on identity formation (Cerezo et al., 2014; Held, 2022; Karimi, 2020b; Lee & Brotman, 2011; Murray, 2014a)		17 %
Migrants found themselves linked with both their old and new social structures, having ties, responsibilities, and feelings towards family and friends in country of origin while resettling (Cerezo et al., 2014; Held, 2022; Karimi, 2020b).	*In the host countries, their lives are far from free, happy and/or safe. In fact, many participants talked about the ambivalence of freedom. For instance, Halim (Germany) described how on one hand he can access more spaces now that feel safe, but that on the other "I miss my home, because I miss my family, I miss the experiences that I had, so it's still this ambivalent feeling. Because I'm here, safe, but safety doesn't mean necessarily feeling happy, or feeling completely satisfied, they don't mean each other, they don't complement each other."* (Held, 2022)	10 %
Conceptualization of sexual orientation and gender identity was influenced by social and cultural forces, and some devoted time and effort to learn about what was necessary to ensure they would appear credible and authentic as LGBTQ+ forced migrants (Lee & Brotman, 2011; Murray, 2014a).	*Sexual minority refugee conceptualizations of sexual orientation and gender identity were fluid and contextual, shifting. and changing over time. This ever shifting relationship between their social location and conceptualization of their sexual and gender identity were profoundly influenced by complex social and cultural forces before, during and after their arrival to Canada.* (Lee & Brotman, 2011)	7 %

**Category: Establishing and maintaining social relationships** (Dhoest, 2019, 2020; Held, 2022; Kahn, 2015a; Karimi, 2020b, 2020a, 2021; Mulé, 2021; Murray, 2014a, 2014b; Oren & Gorshkov, 2021; Wimark, 2019, 2021)		**45 %**
Sub-category: Challenges related to formation of new social relationships (Dhoest, 2019; Held, 2022; Kahn, 2015a; Karimi, 2020a, 2021; Mulé, 2021; Murray, 2014a; Oren & Gorshkov, 2021; Wimark, 2019, 2021)		34 %
Migrants were faced with complex social challenges when trying to manage their communication and forming meaningful relationships, particularly when having limited social contacts in the host country upon arrival (Kahn, 2015a; Karimi, 2021; Mulé, 2021; Murray, 2014a; Wimark, 2019, 2021)	*For all participants, social relationships outside of the family presented on- going challenges after asylum*. (Kahn, 2015a)	21 %
Encountering limited open-mindedness within the general population and communities was a challenge related to forming relationships (Dhoest, 2019; Karimi, 2021)	*The gay men interviewed for this project had limited experi- ence of outright racism in Belgium, but they did comment that it was hard to integrate, partly because they found Belgians not to be very open and open-minded*. (Dhoest, 2019)	7 %
Socioeconomic barriers hindered the formation of social connections between LGBTQ+ migrants and natives (Karimi, 2020a; Mulé, 2021)	*Common points were that these friendships were not as close-knit as friendships with co-ethnic gay men, therefore they were considered to be secondary because "not the same [intimate] as my friendship with my Iranian friends" (Participant 19, 22 years old); and that class boundaries were barely crossed. One participant said "… maybe it is because of where I work or the place that I live… I don't get to know rich people! I'm always among my co-workers or gay guys from around here"*. (Karimi, 2020a)	7 %
Expectations on how to act as an LGBTQ+ person hindered the formation of social connections between LGBTQ+ migrants and natives (Murray, 2014a; Wimark, 2019)	*Tapping into the network of the queer community through dating platforms is, however, not a straightforward endeavour. Like other parts of society, it is laden with values and standards with regard to how to act and be. Being a queer refugee in Sweden can render one different and, to many Swedes, a stranger to be feared*. (Wimark, 2019)	7 %
Lack of interest among non-migrants hindered the formation of social connections between LGBTQ+ migrants and natives (Kahn, 2015a; Wimark, 2019)	*However, connecting with White European or American-born gays in their community was also a com- plicated endeavor: "Most people go through the initial phase of, "Oh, this is so cool." But it's not—it's not cool. It's them trying to look cool, you know, like to prove to themselves that they are open-minded ... like you're exotic animals and we want to come visit you in the zoo, and then it's like, okay, now it's time to go home [Laughs]."* (Kahn, 2015a)	7 %
Migrants balanced interacting with both homophobic and homoaffirmative, as well as coethnic and racist, members within different communities (Kahn, 2015a)	*Jockeying worlds of coethnic versus mainstream, gay versus straight, and homophobic versus homoaffirmative social groups in the quest to build community presented complex challenges*. (Kahn, 2015a)	3 %
Some LGBTQ+ spaces and peer support groups led by white native workers were experienced as permeated by power differentials and oppressive structures therein, further complicating their social situation and potentially increasing alienation (Held, 2022)	*Many of these groups were led, for the most part, by white German/Italian/British LGBTIQ+ workers and volunteers, and although the emotional and practical support LGBTIQ+ claimants and refugees receive in such groups is invaluable, like all spaces, also in LGBTIQ+ (asylum) support spaces, differences and power differentials might exist that shape these spaces in certain ways*. (Held, 2022)	3 %
Another issue with LGBTQ+ organizations experienced by migrants was that they focused too heavily on newcomers and emergency help (Oren & Gorshkov, 2021)	*Though RUSA has been building a community around the LGBTþ rights activism, it also provided practical help such as referrals to attorneys and social services and other information and social events regularly posted on the RUSA website and their Facebook and Meetup pages. Three participants felt disengaged from the RUSA because they thought it focused too much on newcomers and emergency help. They also described a tension between the goals of charity and advocacy*. (Oren & Gorshkov, 2021)	3 %
Sub-category: Communication via the Internet (Dhoest, 2020; Karimi, 2020b; Murray, 2014b; Oren & Gorshkov, 2021; Wimark, 2019, 2021)		21 %
After arrival, migrants gained access to online communication possibilities and information previously less available in their countries of origin (Dhoest, 2020; Karimi, 2020b; Oren & Gorshkov, 2021; Wimark, 2019, 2021)	*After moving to Belgium, the Internet became the key site of information and gay representations for all. For instance, Samuel says, "Coming here, you have a lot of free will to access anything, especially the internet, books, films. I had access to read books, I had access to see movies, I go to gay sites, I can watch movies online, I can watch documentaries online*." (Dhoest, 2020)	17 %

In part, the Internet was used to stay in touch with family members, relatives, LGBTQ+ friends, and the mainstream community in their countries of origin (Dhoest, 2020; Karimi, 2020b; Murray, 2014b)	*Beside information, digital media offer a tool to connect. For instance, if the participants stay in touch with people in their country of origin, they mostly use Internet-based applications such as Skype*. (Dhoest, 2020)	10 %
It was also utilized to communicate with and establish new contacts in the host country, including connecting with LGBTQ+ people to engage in sexual encounters, date, and find a partner (Dhoest, 2020; Wimark, 2019)	*Many interviewees in this project also use chat and dating sites and apps to connect to other gay men. As a separate and more anonymous sphere, it was often a more secure environment for them to first explore their sexuality*. (Dhoest, 2020)	7 %
Sub-category: Gravitating towards certain settings or people (Held, 2022; Karimi, 2021; Wimark, 2019, 2021)		14 %
Some gravitated towards large and diverse cities where they could access LGBTQ+ communities and support (Held, 2022; Wimark, 2019, 2021)	*A sense of freedom and safety was, therefore, dependant on the spatial context and some participants pointed out that while they felt safe when they came to the city, for example, to access LGBTIQ+ support, they did not feel safe in their isolated rural accommodation centres*. (Held, 2022)	10 %
Some gravitated towards rural areas, as densely populated settings induced discomfort and stress (Wimark, 2021)	*The notion of equating being queer with the need to live in a metropolis was a recurrent theme in the interviews with the queer asylum seekers but by far not the only theme. For some, the idea of living in the metropolis was related to discomfort and stress, as the following quotations exemplify: "I live in a quiet area. I have my aunt that lives in [larger city]. So we went there last summer. And it was chaos there, cars, and I couldn't sleep. I couldn't cope because, around nine, maximum at ten, it goes quiet here, that makes me sleep nicely. But there at twelve, one, two o'clock people are on their way still and cars all night. No, I didn't like it. I stayed perhaps two days, and then came back here, I came back here to [rural town], lovely."* (Wimark, 2021)	3 %
Some felt closer to older and other immigrant LGBTQ+ persons because of shared notions and histories of marginalization (Karimi, 2021)	*Further, five participants mentioned that their interactions with the older gen- eration of Canadian gay men and those with immigration backgrounds were less conflictual. After coming to Canada, participants came to believe that what they had seen in movies and romanticized images of LGBT movements and families was closer to what the older Canadian-born gay men had experienced and aspired to in their youth than the indifference and individualist tendencies of younger Canadian gay men*. (Karimi, 2021)	3 %
Sub-category: Moderating disclosure of LGBTQ+ identity (Kahn, 2015a; Karimi, 2020b)		7 %
To keep their family ties and other coethnic relationships intact, some decided to keep a balance between family members and life in host country, moderating what kind of information they share, how much they disclose about their LGBTQ+ identity, and how they justify their emigration (Kahn, 2015a; Karimi, 2020b)	*Interestingly, my informants, whose asylum-seeking decisions were primarily motivated by their sexual orientation and the risks of living in Iran as a gay man, had justified their emigration to their families through the more typical frames of emigration as a search for jobs or education in Turkey and the West. One participant said, "The truth is that I have moved here, but I am still part of my family so from time to time I have to hide who I am and say I immigrated because of work reasons. . . . One of my classmates from Iran got in touch with me asking about universities here in Canada, and now he lives here [Vancouver], and I have to deal with him and the risk of him knowing that I am gay because I do not want him find out and tell others back in Iran."* (Karimi, 2020b)	7 %

References

Akin, D. (2017). Queer asylum seekers: Translating sexuality in Norway. Journal of Ethnic and Migration Studies, 43(3), 458–474. https://doi.org/10.1080/1369183X.2016.1243050

Alessi, E. J. (2016). Resilience in sexual and gender minority forced migrants: A qualitative exploration. Traumatology, 22(3), 203–213. psyh. https://doi.org/10.1037/trm0000077

Alessi, E. J., Greenfield, B., Kahn, S., & Woolner, L. (2021). (Ir)reconcilable identities: Stories of religion and faith for sexual and gender minority refugees who fled from the Middle East, North Africa, and Asia to the European Union. Psychology of Religion and Spirituality, 13(2), 175–183. psyh. https://doi.org/10.1037/rel0000281

Alessi, E. J., Kahn, S., Greenfield, B., Woolner, L., & Manning, D. (2020). A qualitative exploration of the integration experiences of LGBTQ refugees who fled from the Middle East, North Africa, and Central and South Asia to Austria and the Netherlands. Sexuality Research & Social Policy: A Journal of the NSRC, 17(1), 13–26. psyh. https://doi.org/10.1007/s13178-018-0364-7

Alessi, E. J., Kahn, S., Woolner, L., & Van Der Horn, R. (2018). Traumatic Stress Among Sexual and Gender Minority Refugees From the Middle East, North Africa, and Asia Who Fled to the European Union. Journal of Traumatic Stress, 31(6), 805–815. https://doi.org/10.1002/jts.22346

Cerezo, A., Morales, A., Quintero, D., & Rothman, S. (2014). Trans migrations: Exploring life at the intersection of transgender identity and immigration. Psychology of Sexual Orientation and Gender Diversity, 1(2), 170–180. psyh. https://doi.org/10.1037/sgd0000031

Dhoest, A. (2019). Learning to be gay: LGBTQ forced migrant identities and narratives in Belgium. Journal of Ethnic and Migration Studies, 45(7), 1075–1089. Scopus. https://doi.org/10.1080/1369183X.2017.1420466

Dhoest, A. (2020). Digital (dis)connectivity in fraught contexts: The case of gay refugees in Belgium. European Journal of Cultural Studies, 23(5), 784–800. Scopus. https://doi.org/10.1177/1367549419869348

Golembe, J., Leyendecker, B., Maalej, N., Gundlach, A., & Busch, J. (2020). Experiences of Minority Stress and Mental Health Burdens of Newly Arrived LGBTQ Refugees in Germany. Sexuality Research and Social Policy. https://doi.org/10.1007/s13178-020-00508-z*

Held, N. (2022). "As queer refugees, we are out of category, we do not belong to one, or the other": LGBTIQ+ refugees' experiences in "ambivalent" queer spaces. Ethnic and Racial Studies, 1–21. https://doi.org/10.1080/01419870.2022.2032246

Kahn, S. (2015a). Cast out: "Gender Role Outlaws" seeking asylum in the west and the quest for social connections. Journal of Immigrant and Refugee Studies, 13(1), 58–79. Scopus. https://doi.org/10.1080/15562948.2014.894169

Kahn, S. (2015b). Experiences of Faith for Gender Role Non-Conforming Muslims in Resettlement: Preliminary Considerations for Social Work Practitioners. British Journal of Social Work, 45(7), 2038–2055. cin20. https://doi.org/10.1093/bjsw/bcu060

Kahn, S., Alessi, E. J., Kim, H., Woolner, L., & Olivieri, C. J. (2018). Facilitating mental health support for LGBT forced migrants: A qualitative inquiry. Journal of Counseling & Development, 96(3), 316–326. psyh. https://doi.org/10.1002/jcad.12205

Karimi, A. (2020a). Limits of Social Capital for Refugee Integration: The Case of Gay Iranian Male Refugees' Integration in Canada. International Migration, 58(5), 87–102. Scopus. https://doi.org/10.1111/imig.12691

Karimi, A. (2020b). Refugees' Transnational Practices: Gay Iranian Men Navigating Refugee Status and Cross-border Ties in Canada. Social Currents, 7(1), 71–86. Scopus. https://doi.org/10.1177/2329496519875484

Karimi, A. (2021). Sexuality and integration: A case of gay Iranian refugees' collective memories and integration practices in Canada. Ethnic and Racial Studies, 44(15), 2857–2875. https://doi.org/10.1080/01419870.2018.1550207

Kostenius, C., Hertting, K., Pelters, P., & Lindgren, E.-C. (2021). From Hell to Heaven? Lived experiences of LGBTQ migrants in relation to health and their reflections on the future. Culture, Health & Sexuality, 1–13. https://doi.org/10.1080/13691058.2021.1983020

Lee, E. O. J., & Brotman, S. (2011). Identity, refugeeness, belonging: Experiences of sexual minority refugees in Canada. Canadian Review of Sociology = Revue Canadienne de Sociologie, 48(3), 241–274. https://doi.org/10.1111/j.1755-618x.2011.01265.x

Llewellyn, C. (2021). Captive While Waiting to Be Free: Legal Violence and LGBTQ Asylum Applicant Experiences in the USA. Sexuality Research and Social Policy, 18(1), 202–212. Scopus. https://doi.org/10.1007/s13178-020-00449-7

Logie, C. H., Lacombe-Duncan, A., Lee-Foon, N., Ryan, S., & Ramsay, H. (2016). "It's for us -newcomers, LGBTQ persons, and HIV-positive persons. You feel free to be": A qualitative study exploring social support group participation among African and Caribbean lesbian, gay, bisexual and transgender newcomers and refugees in Toronto, Canada. BMC International Health and Human Rights, 16(1), 18. https://doi.org/10.1186/s12914-016-0092-0

Mulé, N. J. (2021). Mental health issues and needs of LGBTQ+ asylum seekers, refugee claimants and refugees in Toronto, Canada. Psychology and Sexuality. Scopus. https://doi.org/10.1080/19419899.2021.1913443

Murray, D. A. B. (2014a). Real queer: "Authentic" LGBT refugee claimants and homonationalism in the Canadian Refugee System. Anthropologica, 56(1), 21–32. psyh.

Murray, D. A. B. (2014b). The challenge of home for sexual orientation and gendered identity refugees in Toronto. Journal of Canadian Studies, 48(1), 132–152. Scopus. https://doi.org/10.1353/jcs.2014.0019

Novitskaya, A. (2021). Sexual Citizens in Exile: State-Sponsored Homophobia and Post-Soviet LGBTQI+ Migration. Russian Review, 80(1), 56–76. Scopus. https://doi.org/10.1111/russ.12298

Oren, T., & Gorshkov, A. (2021). Lived Experiences of Recent Russian-Speaking LGBT+ Immigrants in the United States: An Interpretive Phenomenological Analysis. Journal of LGBT Issues in Counseling, 15(3), 290–309. cin20. https://doi.org/10.1080/15538605.2021.1914278

Rosati, F., Coletta, V., Pistella, J., Scandurra, C., Laghi, F., & Baiocco, R. (2021). Experiences of Life and Intersectionality of Transgender Refugees Living in Italy: A Qualitative Approach. International Journal of Environmental Research and Public Health, 18(23). https://doi.org/10.3390/ijerph182312385

Wimark, T. (2019). Homemaking and perpetual liminality among queer refugees. Social and Cultural Geography. Scopus. https://doi.org/10.1080/14649365.2019.1619818

Wimark, T. (2021). Housing policy with violent outcomes–the domestication of queer asylum seekers in a heteronormative society. Journal of Ethnic and Migration Studies, 47(3), 703–722. Scopus. https://doi.org/10.1080/1369183X.2020.1756760
